# Manifestations of intraocular inflammation over time in patients on brolucizumab for neovascular AMD

**DOI:** 10.1007/s00417-021-05518-0

**Published:** 2021-12-21

**Authors:** Ramin Khoramnia, Marta S. Figueroa, Lars-Olof Hattenbach, Carlos E. Pavesio, Majid Anderesi, Robert Schmouder, Yu Chen, Marc D. de Smet

**Affiliations:** 1 Department of Ophthalmology, The David J. Apple Center for Vision Research, Heidelberg University Hospital, Im Neuenheimer Feld 400, 69120 Heidelberg, Germany; 2 Department of Ophthalmology, International Vision Correction Research Centre, Heidelberg University Hospital, Im Neuenheimer Feld 400, 69120 Heidelberg, Germany; 3 Retina Division, Ramón y Cajal University Hospital, Madrid, Spain; 4 Department of Ophthalmology, Ludwigshafen Hospital, Ludwigshafen am Rhein, Germany; 5 Department of Uveitis, Moorfields Eye Hospital and UCL, London, UK; 6 Novartis Pharma AG, Basel, Switzerland; 7 Novartis Pharmaceuticals Corporation, East Hanover, NJ USA; 8 Medical/Surgical Retina and Ocular Inflammation, Microinvasive Ocular Surgery Center (MIOS Sa), Lausanne, Switzerland

**Keywords:** Intraocular inflammation, Retinal vasculitis, Retinal vascular occlusion, Anti-vascular endothelial growth factor, Brolucizumab

## Abstract

**Purpose:**

To describe the adverse events associated with brolucizumab, in particular the sequence of intraocular inflammation (IOI), retinal vasculitis (RV), and/or retinal vascular occlusion (RO).

**Methods:**

This was an unmasked post hoc analysis of the randomized HAWK/HARRIER clinical trials. Patients with neovascular AMD in the brolucizumab arms of the trials were included. IOI-related adverse events reported by study investigators were analyzed to determine early signs and the time course of IOI-related adverse events, using a subgroup of patients with definite/probable IOI cases identified in an independent unmasked post hoc review by an external safety review committee. A limited literature review on IOI following anti-VEGF therapy was also conducted.

**Results:**

Among 50 patients with definite/probable IOI cases identified by the safety review committee, 12 had RV or RO adverse events reported by the investigators. For 6 of 12, IOI (other than RV) was reported before RV or RO. The duration from the first IOI adverse event to the first RV or RO adverse event ranged from 16 to 171 days for 5 patients and was 553 days for 1 patient. Four of the 6 patients received ≥ 1 brolucizumab injection on or after the date of the first IOI adverse event and before the first RV or RO adverse event.

**Conclusions:**

IOI may precede RV or RO in some patients treated with brolucizumab.

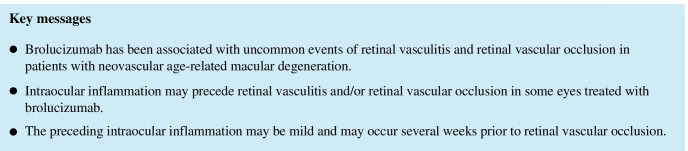

**Supplementary Information:**

The online version contains supplementary material available at 10.1007/s00417-021-05518-0.

## Introduction

Intravitreal injection of anti-vascular endothelial growth factor (anti-VEGF) agents is generally well tolerated in the treatment of neovascular age-related macular degeneration (nAMD) [1]. Following anti-VEGF treatment, intraocular inflammation (IOI) has been found to occur at low rates [2]. In the pivotal nAMD trials for the approved anti-VEGF drugs, rates of IOI were reported to be ≤ 2.1% in ANCHOR and MARINA (any ocular inflammation of grade 3 or 4), ≤ 1.5% in VIEW 1 and VIEW 2, and ≤ 4.7% in HAWK and HARRIER [3–6]. IOI following anti-VEGF treatment often manifests as presence of cells in the anterior chamber or vitreous with reduced or blurred vision, which is typically treated with topical steroids and generally resolves without complication [2, 7–12]. Since FDA approval of brolucizumab in October 2019, there have been post-marketing reports of events of retinal vasculitis (RV) and retinal vascular occlusion (RO) associated with IOI following administration of brolucizumab [13, 14].

Brolucizumab is a humanized, single-chain antibody fragment that inhibits all isoforms of VEGF-A [15, 16]. The molecule is composed of the antibody variable light and variable heavy chain domains linked together by a flexible peptide, with complementarity-determining regions providing high affinity to VEGF [17]. At approximately 26 kDa, it is the smallest of the anti-VEGF molecules clinically available (ranibizumab: 48 kDa; aflibercept: 115 kDa; and bevacizumab: 147 kDa) [15, 17]. The biophysical properties of brolucizumab allow for a high concentration of the drug to be delivered per intravitreal injection, at 6 mg/50 µL [15, 17, 18]. The smaller size and high concentration are hypothesized to allow for a high degree of ocular tissue penetration [16, 19].

In the pivotal HAWK and HARRIER studies, brolucizumab administered every 8 to 12 weeks was found to be noninferior to aflibercept administered every 8 weeks in visual acuity gains at week 48, with 55.6% and 51.0% of patients maintained on a 12-week dosing regimen in HAWK (6 mg) and HARRIER, respectively [19]. Brolucizumab demonstrated superiority over aflibercept in reduction in central subfield thickness and rates of retinal fluid at week 48 (e.g., least squares mean change from baseline in central subfield thickness was − 172.8 µm vs − 143.7 µm for brolucizumab 6 mg vs aflibercept in HAWK; *P* = 0.001) [19]. These visual acuity gains and anatomical findings were maintained out to week 96 [6].

When the safety results of the HAWK and HARRIER studies were evaluated, the rate of IOI adverse events was higher in the brolucizumab 6 mg arm than in the aflibercept arm up to week 48 (HAWK: 5.3% vs 0.3%; HARRIER: 2.7% vs 0.8%) [17]. Rates of vision loss (≥ 15-letter loss from baseline) were similar between the brolucizumab (6 mg) and aflibercept groups at week 48 (HAWK: 6.4% vs 5.5%; HARRIER: 3.8% vs 4.8%) [19]. RV and RO were defined by various *Medical Dictionary for Regulatory Activities* (MedDRA) preferred terms, including *retinal vasculitis*, and *retinal perivascular sheathing*, *retinal artery occlusion*, *retinal artery embolism*, *retinal artery thrombosis*. The association between brolucizumab and RV and RO adverse events was not identified until post-approval cases of occlusive retinal vasculitis were reported [13, 19]. In response to the reports, Novartis established an external safety review committee (SRC) to provide an independent, unmasked post hoc review of IOI, endophthalmitis, and retinal artery occlusion in the HAWK and HARRIER studies [13, 20]. Based on a review of images of the brolucizumab-treated eye of 60 patients with IOI, retinal artery occlusion, and/or endophthalmitis, the SRC concluded that 50 patients (4.6%; 50/1088) of the 1088 patients in the brolucizumab arms had definite or probable IOI. Of these 50, 36 (3.3%; 36/1088) also experienced RV; and of these 36, 23 (2.1%; 23/1088) also experienced RO. The estimated overall rate of definite/probable IOI with vision loss ≥ 15 letters from baseline in the brolucizumab arms was 0.7% (8/1088). Based on post-marketing reports, the reporting rate of RV and/or RO is 15.5 per 10,000 injections, as of July 30, 2021 [21].

The goal of the present analysis was to describe the sequence of clinical events reported by the study investigators in the 50 patients with cases of IOI identified by the SRC to determine the early signs and time course of the adverse events, especially whether IOI (other than RV) precedes RV or RO. A finding that IOI precedes RV or RO would suggest that IOI can be used as an early sign that a patient may be at risk of RV or RO, which could allow for early monitoring and management. A second goal of the present analysis was to summarize signs and symptoms of IOI related to brolucizumab as reported in the literature.

## Methods

Novartis established a coalition consisting of a dedicated internal team to collaborate with top global experts to examine potential root causes, risk factors, mitigation, and potential treatment protocols for adverse events associated with brolucizumab. Using the patients identified by the SRC review as a starting point, the authors of this manuscript conducted an unmasked post hoc analysis of the study-investigator reports to determine early signs and time course of IOI-related adverse events. The authors also conducted a limited literature review on IOI following anti-VEGF therapy to supplement the post hoc analysis.

### Literature review

The literature review was conducted to identify clinical signs and symptoms of noninfectious IOI following anti-VEGF therapy in nAMD. It was based on a PubMed search conducted on August 28, 2020, using 88 clinical terms based on the MedDRA preferred terms and ICD-10-CM codes (search terms are provided in the Online Resource 1). Relevant articles based on bibliographies and open search were also included. Only full-text journal articles in English of primary research describing signs and symptoms of IOI following anti-VEGF therapy in nAMD were considered for the review. Articles describing infectious or presumed-infectious endophthalmitis as the main form of inflammation were excluded, because infectious endophthalmitis was considered distinct from IOI and sterile endophthalmitis.

### Post hocanalysis

The HAWK and HARRIER studies were 2-year, phase 3, double-masked, randomized trials conducted at 408 sites (ClinicalTrials.gov identifier NCT02307682, NCT02434328) [19]. Eligible patients were ≥ 50 years of age and had untreated, active choroidal neovascularization secondary to AMD [19]. Full details on the methods of HAWK and HARRIER have been described previously [19]. All patients provided written informed consent. Approval was obtained by an independent ethics committee or an institutional review board, and the studies complied with the ethical standards of the Declaration of Helsinki. For the present post hoc analysis of the HAWK and HARRIER studies, the 2-year safety analysis set was used, including only the patients who were treated with brolucizumab (3 mg or 6 mg in HAWK and 6 mg in HARRIER).

The present study analyzed ocular adverse events based on the report of the study investigators in HAWK and HARRIER for the subgroup of patients with definite/probable IOI cases identified by the SRC (*n* = 50). The identification of patients with IOI cases by the SRC has been previously described [20]. The SRC reviewed the images of the brolucizumab-treated eye of 60 patients with IOI, retinal artery occlusion, and/or endophthalmitis reported by the study investigators. Of the 60 patients, the SRC excluded 10 patients with adverse events that, in their opinion, were “not drug-related, not in spectrum.” For the subgroup of 50 patients with definite/probable IOI cases, ocular adverse events were analyzed in the present study based on the report of the study investigators in HAWK and HARRIER (using various MedDRA preferred terms) to allow for the analysis of the time course of individual adverse events of IOI and RV or RO. Notably, the number of patients with RV or RO in the present analysis is lower than the number of patients with RV or RO identified by the SRC (*n* = 36), because the study investigators did not observe all the events of RV and RO that the SRC observed. To distinguish between events identified by the study investigators and patients identified by the SRC in the present study, the term *adverse event* is used to describe events detected by the study investigators during the conduct of HAWK and HARRIER, and the term *case* is used when referring to patients identified by the SRC (e.g., “patient with an IOI case”).

IOI-related adverse events were defined in the present analysis as events of IOI, RO, or endophthalmitis reported by investigators in the HAWK and HARRIER trials. Ocular MedDRA preferred terms used to define IOI adverse events are provided in Online Resource 2 and included terms also considered RV (*retinal vasculitis* and *retinal perivascular sheathing*) unless otherwise specified. Ocular MedDRA preferred terms used to define RO adverse events are provided in Online Resource 3. MedDRA preferred terms used to define RV and/or RO collectively included *retinal vasculitis* and *retinal perivascular sheathing* and the terms in Online Resource 3.

For each patient with an IOI case (*n* = 50), the time from the first injection of brolucizumab to the onset of the first IOI-related adverse event (onset date − first injection date + 1), the time between the preceding injection and the onset of the first IOI-related adverse event (onset date − the last injection date before the onset date), and the number of injections before the onset of the first IOI-related adverse event were calculated. For patients with RV or RO adverse events who showed an IOI adverse event (other than RV) preceding the RV or RO event, the time between the first IOI event and the first event of RV or RO was calculated (first RV or RO onset date − first IOI onset date + 1). The number of injections on or after the onset of the first IOI adverse event and before the first adverse event of RV or RO was also calculated.

In addition, ocular adverse events other than those used to define IOI and RO that were thought to occur more frequently in patients with IOI were identified from a list of all ocular adverse events (reported using MedDRA preferred terms) that occurred in the HAWK and HARRIER trials. Rates of these ocular adverse events were compared between patients with and without IOI cases, and the rates of these adverse events prior to the first IOI-related adverse events in patients with IOI cases were calculated. No inferential testing was planned or conducted for group comparison. SAS version 9.4 or higher was used to provide descriptive statistics.

## Results

### Literature review

The literature search identified 975 articles. The abstracts and titles were screened to exclude records that were not in English, nonprimary research, health economics and outcomes research, reports on indications not including nAMD, and reports on drug therapies not including anti-VEGF therapy. Two hundred forty-six records remained. These were further screened to exclude records that did not describe signs and symptoms of IOI and those that described infectious endophthalmitis. This resulted in 31 articles that were reviewed. Twenty-six of the 31 articles involved anti-VEGF agents other than brolucizumab. These 26 did not report retinal vasculitis [2, 7, 22–24]. Five of the 31 records described post-marketing studies of RV or RO following administration of brolucizumab and were deemed most relevant for the present literature review [25–29]. It is unknown whether there was overlap between the patients described in these studies.

Three of the 5 records describing brolucizumab-associated RV or RO were case studies. The other 2 records were retrospective studies, including 26 eyes in 25 patients and 15 eyes in 12 patients, respectively [25–29]. In all 5 studies, patients were switched to brolucizumab from a different anti-VEGF and had received 1 to 3 injections of brolucizumab before the adverse event [25–29]. Witkin et al. found that the mean time to presentation of the adverse event from the preceding brolucizumab injection was 26 days (range = 3–36) among 26 eyes [25]. Similarly, Baumal et al. found that the time to symptom development from the preceding brolucizumab injection ranged from 3 to 56 days among 15 eyes, while the mean time from the preceding brolucizumab injection to diagnosis of RV was 30.3 days (range = 14–56) [26]. Among the 3 case studies, the times from the preceding brolucizumab injections to presentation of the adverse event were within these ranges [27–29].

Symptoms reported in association with RV or RO following brolucizumab injection primarily included reduced or blurry vision, floaters, pain, redness, scotoma, and ocular discomfort [25–29]. Witkin et al. reported the incidence (*N* = 26) of symptoms at the onset of the adverse event to be 62% for blurry vision, 46% for floaters, 31% for pain, 19% for redness, and 12% for scotoma; 8% were asymptomatic [25]. Baumal et al. observed that all patients reported at least 1 of the following symptoms at the onset of the adverse event: floaters (8/12), reduced or blurred vision (7/12), scotoma (3/12), and ocular discomfort (2/12) [26]. In both studies, reduced or blurred vision and floaters were relatively common symptoms. In some patients, floaters and reduced vision were found to precede evidence of RV [26]. Other symptoms reported at the onset of the adverse event have been foreign body sensation, light sensitivity, flashes, and dimming of visual acuity [26, 28]. Most of these symptoms, aside from scotoma and flashes, are symptoms reported to occur with IOI following administration of other anti-VEGFs [2, 7–9, 11, 22–24, 30, 31].

Signs of IOI (other than RV) associated with RV or RO following brolucizumab include inflammatory cells in the anterior and/or posterior segments. Witkin et al. reported that signs of IOI (other than RV) were present in 92% of eyes with RV [25]. The location of inflammatory cells was anterior only in 31%, posterior only in 27%, or both anterior and posterior in 35% [25]. In 5 of 22 eyes with occlusive RV, the occlusive RV developed following signs of IOI (without RV); in 1 of 22 eyes, the occlusive RV developed following nonocclusive RV [25]. Baumal et al. reported that vitreous cells or opacities were present in all 15 eyes with RV, and 11 of 15 eyes showed anterior chamber cells [26]. In addition, nongranulomatous keratic precipitates, conjunctival injection, Descemet’s folds, and corneal edema were clinical features associated with RV following brolucizumab injection [26].

Brolucizumab-associated vascular involvement has been described as varying in severity, depending on presence or absence of occlusive vasculitis and on the location of the occlusions [25]. Vascular involvement with brolucizumab may affect the macula and/or the peripheral retina and may affect retinal arteries, retinal veins, and/or choroidal vessels, with retinal arteries seeming to be affected most commonly [25, 26]. Baumal et al. found that 6 of 12 patients showed occlusions in large retinal arteries near the optic nerve or fovea, while 5 of 12 patients showed occlusions or filling defects farther from the fovea, and 1 of 12 patients showed only perivenular sheathing, demonstrating the range of severity of vascular involvement with brolucizumab [26]. Although the findings from these post-marketing studies are limited by low numbers of patients, the overall findings suggest that patients with RV or RO following brolucizumab treatment typically have signs of IOI in addition to RV. In some cases, IOI or symptoms associated with IOI were found to precede RV [25, 26]. Signs of IOI may therefore be useful for early detection of the spectrum of IOI, RV, and/or RO in patients treated with brolucizumab.

### Post hocanalysis of HAWK and HARRIER

#### Patients with and without intraocular inflammation cases

The SRC identified 50 patients with definite/probable IOI cases following treatment with brolucizumab among the 60 patients included in the SRC review. A higher proportion of patients with definite/probable IOI cases (*n* = 50) than without IOI cases (*n* = 1038) discontinued brolucizumab treatment early in the HAWK and HARRIER studies (52.0% [26/50] vs 13.8% [143/1038]). In addition, a higher proportion of patients with definite/probable IOI cases than without IOI cases discontinued the study early (24.0% [12/50] vs 11.6% [120/1038]). The mean number of brolucizumab injections received was 7.5 (SD = 3.9) for patients with IOI cases and 10.7 (SD = 2.4) for patients without IOI cases.

**Fig. 1:**
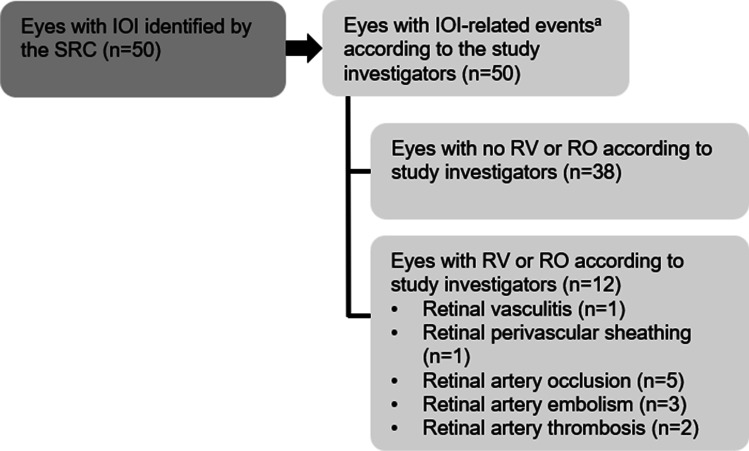
Diagram of the patients included in the analysis. Patients included were those 50 patients with definite/probable intraocular inflammation (IOI) as identified by the safety review committee (SRC). According to the study investigators, these patients each had ≥ 1 IOI-related adverse event. ^a^IOI-related adverse events included IOI, endophthalmitis, or retinal vascular occlusion (RO). IOI, intraocular inflammation; RO, retinal vascular occlusion; RV, retinal vasculitis; SRC, safety review committee

Of the 50 patients with definite/probable IOI cases, each patient had ≥ 1 IOI-related adverse event, according to the study investigators. Table 1 shows the number of patients with IOI (excluding RV), endophthalmitis, RV, and RO and the severity of these IOI-related adverse events. At the adverse event level, there were 63 IOI adverse events (excluding RV adverse events), 5 endophthalmitis adverse events, 2 RV adverse events, and 11 RO adverse events, according to the study investigators. As illustrated in Fig. 1, 12 of the 50 patients were reported to have RV or RO adverse events, according to the study investigators. Among these 12 patients, the MedDRA preferred terms describing the RV or RO adverse events were retinal vasculitis (1/12), retinal perivascular sheathing (1/12), retinal artery occlusion (5/12), retinal artery embolism (3/12), or retinal artery thrombosis (2/12; Online Resource 4). Among the patients with RV and RO adverse events, 2 patients had serious retinal artery occlusion, 1 patient had serious retinal artery embolism, and 2 patients had serious retinal artery thrombosis. Five of the 12 patients with RV or RO had at least moderate vision loss (≥ 15 ETDRS letters) at the end of the study. For those patients with IOI cases who also discontinued brolucizumab treatment early, the number of days and injections between the onset of the first IOI-related adverse event and discontinuation of brolucizumab treatment is summarized in Online Resource 5 and Online Resource 6.

**Table 1:** IOI-related adverse event severity. The number of patients with at least 1 adverse event in each adverse event category, and the number of patients with a maximum severity for the adverse event category at each severity level in patients with definite/probable intraocular inflammation cases (*N* = 50)^a^

Adverse event category	Total	Mild^b^	Moderate^c^	Severe^d^
Any IOI, RO, or endophthalmitis	50	19	23	8
Endophthalmitis	5	1	3	1
IOI (excluding RV)	43	19	20	4
RV	2	1	1	0
RO	10	3	2	5
IOI, RO, or endophthalmitis reported at the IOI onset	50	25	21	4
Endophthalmitis	4	1	2	1
IOI (excluding RV)	41	21	17	3
RV	2	1	1	0
RO	4	2	1	1

^a^A patient with multiple occurrences or multiple severity ratings of an adverse event for a preferred term or category was counted only once under the maximum rating in each category

^b^*Mild* was defined as usually transient in nature and generally not interfering with normal activities

^c^*Moderate* was defined as sufficiently discomforting to interfere with normal activities

^d^*Severe* was defined as preventing normal activities

*IOI*, intraocular inflammation; *RV*, retinal vasculitis; *RO*, retinal vascular occlusion

#### Timing of the intraocular inflammation–related adverse events relative to brolucizumab injections

**Fig. 2:**
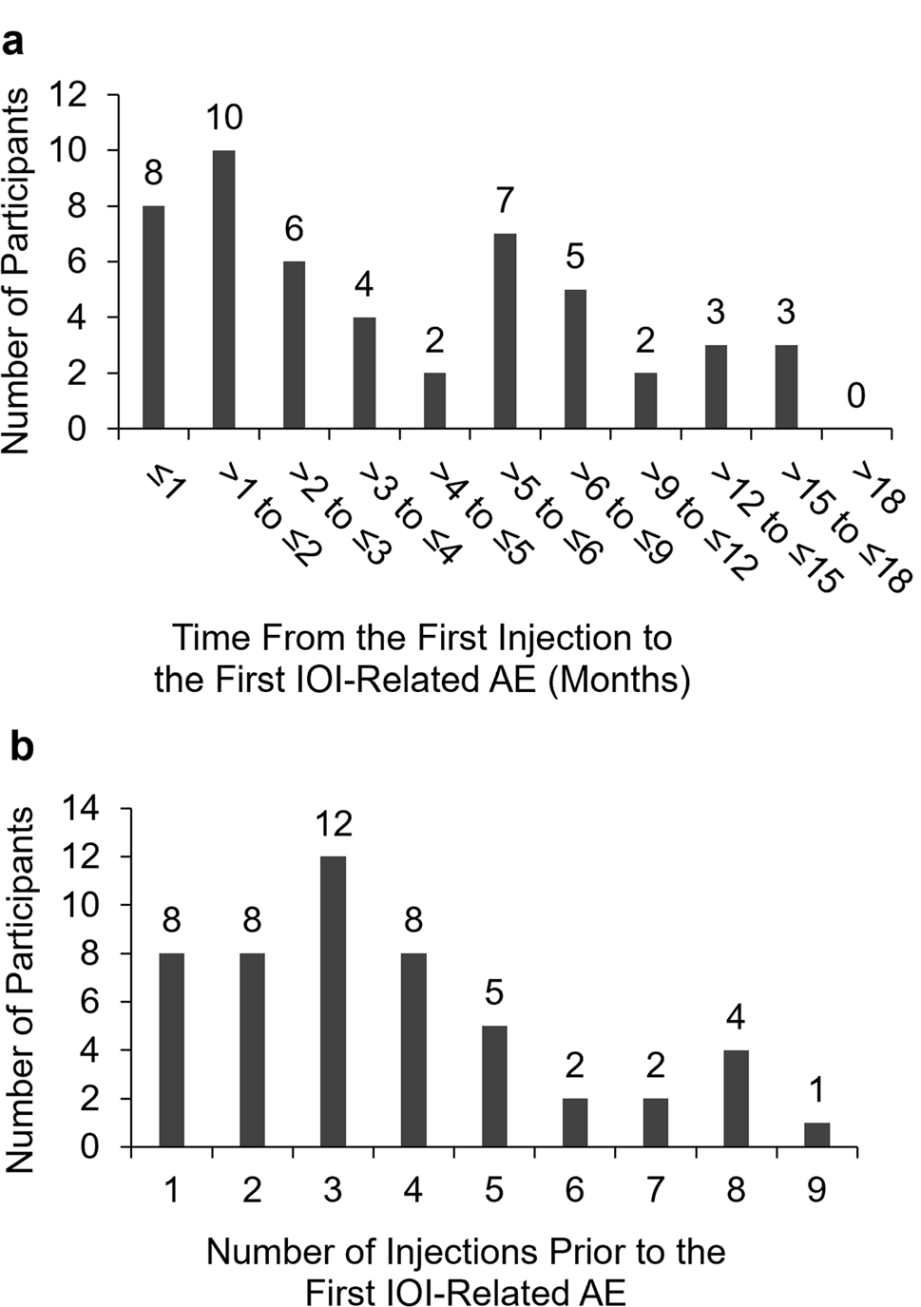
Time from the first study injection to the onset of the first intraocular inflammation–related adverse event for patients with definite/probable intraocular inflammation cases (*N* = 50). **a** The frequency distribution of the time (months) from the first intravitreal injection of the study treatment (brolucizumab 3 mg or 6 mg) to the onset of the first intraocular inflammation (IOI)–related adverse event (AE) in the HAWK and HARRIER studies. **b** The frequency distribution of the number of injections before the first IOI-related AE. AE, adverse event; IOI, intraocular inflammation

The time from the first brolucizumab injection to the onset of the first IOI-related adverse event ranged from less than a month to over 15 months (Fig. 2a). For almost half of patients (48.0%; 24/50), the first IOI-related adverse event was detected in the first 3 months of treatment (i.e., during the loading-dose phase). For 8 of these patients (16%; 8/50), the first IOI-related adverse event was detected in the first month of treatment. For approximately a quarter of patients (26.0%; 13/50), the first IOI-related adverse event was detected in months 4 to 6 (> 3 months to ≤ 6 months); for approximately another quarter (26.0%; 13/50), the first IOI-related adverse event was detected in months 7 to 18 (> 6 months to ≤ 18 months). In terms of the number of injections before the onset of the first IOI-related event, 56.0% (28/50) of patients received 1 to 3 injections before the onset of the adverse event. Approximately a quarter (26.0%; 13/50) received 4 or 5 injections before the onset of the adverse event, and 18.0% (9/50) received 6 or more injections before the onset of the adverse event (Fig. 2b).

**Fig. 3:**
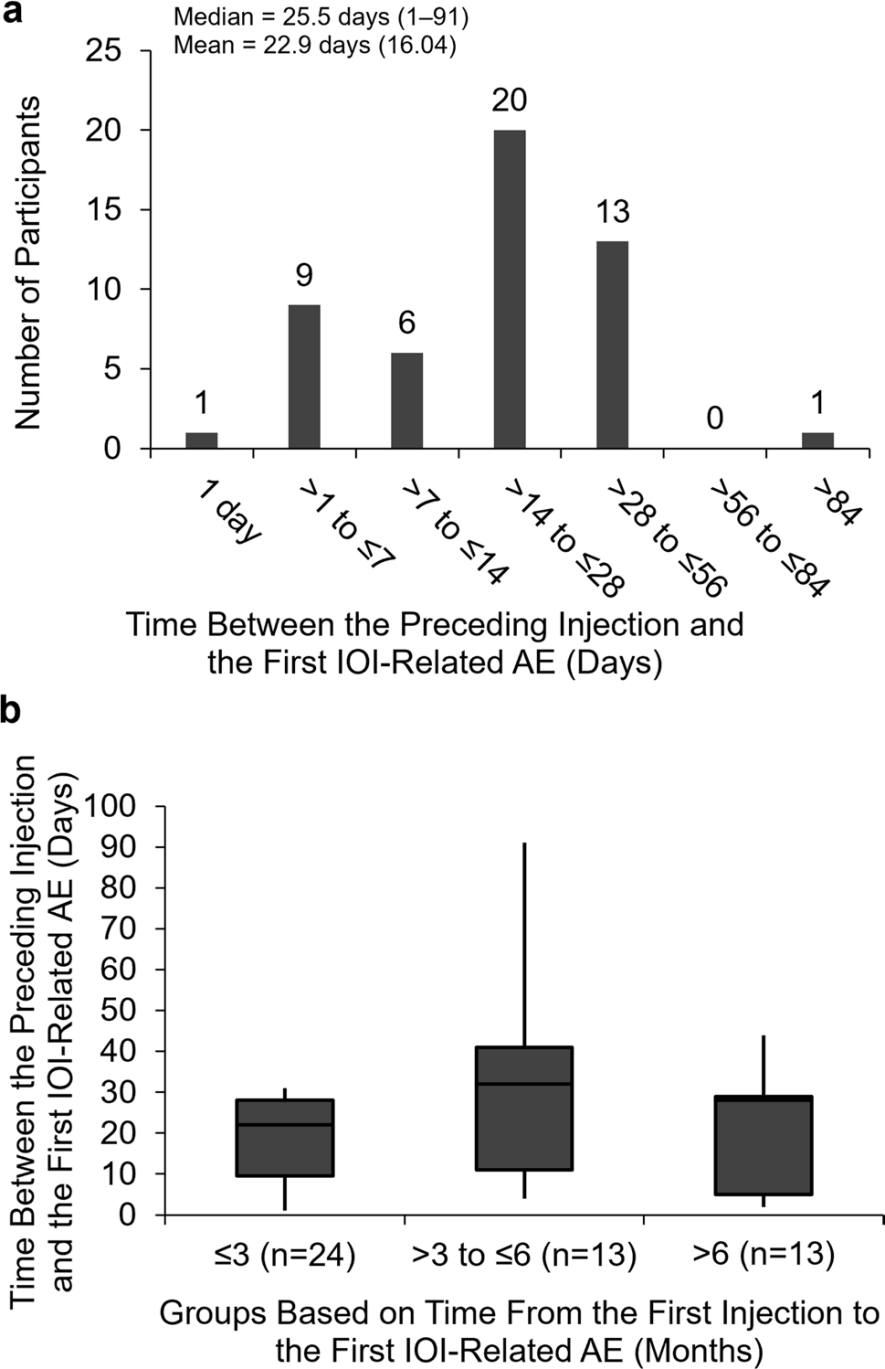
Time between the preceding study injection and the onset of the first intraocular inflammation–related adverse event in patients with definite/probable intraocular inflammation cases (*N* = 50). **a** The frequency distribution of the time (days) between the preceding intravitreal injection of the study treatment (brolucizumab 3 mg or 6 mg) and the onset of the first intraocular inflammation (IOI)–related adverse event (AE) in the HAWK and HARRIER studies. Median (range) and mean (SD) are reported. **b** The time (days) between the preceding intravitreal injection of the study treatment and the onset of the first IOI-related AE for subgroups defined by the time from the first study treatment to the onset of the first IOI-related AE (months). The boxes show the interquartile range. The whiskers extend to the minimum and maximum values. AE, adverse event; IOI, intraocular inflammation

The time between the preceding brolucizumab injection and the onset of the first IOI-related adverse event is shown in Fig. 3a. The median time between the preceding brolucizumab injection and the onset of the first IOI-related adverse event was 25.5 days (range = 1–91 days; mean = 22.9 [SD = 16.04]). For 72.0% (36/50) of patients, the first IOI-related adverse event was detected ≤ 28 days after the preceding injection, and for 98.0% (49/50) of patients, the first IOI-related adverse event was detected ≤ 56 days after the preceding injection. When the time to onset of the first IOI-related adverse event from the first injection was considered (Fig. 3b), the maximum time between the preceding brolucizumab injection and the onset of the first IOI-related adverse event for patients diagnosed in the first 3 months of treatment (≤ 3 months) was 31 days (median = 22 days [range = 1–31; interquartile range = 9.5–28.0]), which was an upper limit because of the monthly schedule of the first 3 injections. For patients first diagnosed with an IOI-related adverse event in months 4 to 6 (> 3 to ≤ 6 months) and month 7 or later (> 6 months), the median time between the preceding injection and the onset of the first IOI-related adverse event was 32 days (range = 4–91; interquartile range = 11.0–41.0) and 28 days (range = 2–44; interquartile range = 5.0–29.0), respectively. The median time between the preceding injection and the first RV or RO adverse event was 29.5 days (range = 6–594; interquartile range = 19–42; n = 12).

#### Brolucizumab injections received during intraocular inflammation and endophthalmitis adverse events

Among the 50 patients with definite/probable IOI cases, there were 70 IOI (including RV) or endophthalmitis adverse events according to the study investigators. Despite the contraindication of administering brolucizumab during active IOI, for 38.6% (27/70) of IOI or endophthalmitis adverse events, ≥ 1 brolucizumab injection was administered on or after the onset of the adverse event and on or before the end date of the adverse event. For 11.4% (8/70) of IOI or endophthalmitis adverse events, ≥ 2 brolucizumab injections were administered during this interval.

#### First presenting intraocular inflammation–related adverse events

For the 50 patients with definite/probable IOI cases, the first presenting IOI-related adverse events most commonly included uveitis (26.0%), iritis (14.0%), and iridocyclitis (10.0%; Table 2). For 6 of 12 patients with investigator-reported RV or RO adverse events, RV or RO was the first IOI-related adverse event (retinal vasculitis [1/6], perivascular sheathing [1/6], retinal artery occlusion [3/6], retinal artery embolism [1/6]). For the other 6 with investigator-reported RV or RO adverse events, an IOI adverse event (other than RV) was reported before the first RV or RO (retinal artery occlusion [2/6], retinal artery embolism [2/6], retinal artery thrombosis [2/6]). The medications administered and outcomes of the first IOI-related adverse events are listed in Online Resource 7.

**Table 2:** Incidence of the first presenting intraocular inflammation–related adverse events^a^ in patients with definite/probable intraocular inflammation cases

First IOI-related adverse event^b,c^	Patients with IOI cases (*N* = 50), *n* (%)
Uveitis	13 (26.0)
Iritis	7 (14.0)
Iridocyclitis	5 (10.0)
Endophthalmitis	4 (8.0)
Anterior chamber inflammation	3 (6.0)
Chorioretinitis	3 (6.0)
Retinal artery occlusion	3 (6.0)
Vitritis	3 (6.0)
Anterior chamber cell	2 (4.0)
Eye inflammation	2 (4.0)
Keratic precipitates	2 (4.0)
Anterior chamber flare	1 (2.0)
Retinal artery embolism	1 (2.0)
Retinal perivascular sheathing	1 (2.0)
Retinal vasculitis	1 (2.0)

^a^Intraocular inflammation (IOI)–related adverse events were defined as IOI, retinal vascular occlusion, or endophthalmitis

^b^A patient may have had more than 1 adverse event and could occur in more than 1 row

^c^Preferred terms based on the *Medical Dictionary for Regulatory Activities* version 20.1 were used for reporting

*IOI*, intraocular inflammation

Table 3 describes the IOI-related adverse events for the 6 patients with IOI adverse events preceding RV or RO adverse events. The preceding IOI adverse events were reported for each of the 6 patients as iridocyclitis (2/6), iritis (1/6), uveitis or uveitis and vitreous floaters (2/6), and vitritis (1/6). The preceding IOI was considered mild for 3 of 6 patients (the most severe preceding IOI adverse event is represented for patients with > 1 preceding IOI adverse event). The time between the onset of the first IOI and the onset of the first RV or RO ranged from 16 to 171 days for 5 of 6 patients. For 1 of 6, the onset of the first RV or RO occurred 553 days after the onset of the first IOI. For this patient, brolucizumab was discontinued upon detection of the IOI. Further investigation revealed that the RO was described as a Hollenhorst plaque not associated with vitreal or aqueous cells. Four of 6 patients received ≥ 1 brolucizumab injection on or after the onset of the first IOI adverse event and before the first RV or RO adverse event. Of the 6 patients with RV or RO adverse events and preceding IOI adverse events, 3 had at least moderate vision loss (≥ 15 ETDRS letters).

**Table 3:** Patients with intraocular inflammation adverse events (other than retinal vasculitis) before retinal vasculitis or retinal vascular occlusion adverse events of the patients with definite/probable intraocular inflammation cases (*N* = 50). Details on the preceding intraocular inflammation (IOI) and the first retinal vasculitis (RV) or retinal vascular occlusion (RO) in the 6 patients (of 12 total patients with RV or RO adverse events) who showed an IOI adverse event (other than RV) before an RV or RO adverse event

Subject	Preceding IOI (preferred term)^a^	Severity of preceding IOI^b,c^	First RV or RO adverse event (Preferred term)^a^	Severity of RV or RO	Number of days between the first IOI and first RV or RO^d^	Number of injections between the first IOI and first RV or RO^e^	Moderate vision loss (≥ 15 ETDRS letters)
4	Iridocyclitis; iridocyclitis	Severe	Retinal artery occlusion	Severe	43	1	Yes
24	Iritis	Mild	Retinal artery embolism	Moderate	171	3	No
29	Uveitis; vitreous floaters	Moderate	Retinal artery thrombosis	Severe	16	0	Yes
32	Vitritis	Moderate	Retinal artery embolism	Mild	553	0	No
41	Uveitis	Mild	Retinal artery occlusion	Mild	36	1	No
42	Iridocyclitis	Mild	Retinal artery thrombosis	Severe	22	1	Yes

^a^Preferred terms based on the *Medical Dictionary for Regulatory Activities* version 20.1 were used for reporting

^b^The severity reported is that of the most severe IOI adverse event for those patients with > 1 IOI adverse event preceding the RV or RO adverse event

^c^*Mild* was defined as usually transient in nature and generally not interfering with normal activities. *Moderate* was defined as sufficiently discomforting to interfere with normal activities. *Severe* was defined as preventing normal activities

^d^Number of days was calculated as the onset date of the first RV or RO—the onset date of the first IOI + 1

^e^The number of injections is the total number of injections on or after the onset date of the first IOI adverse event and before the first RV or RO adverse event

*ETDRS*, Early Treatment Diabetic Retinopathy Study; *IOI*, intraocular inflammation; *RO*, retinal vascular occlusion; *RV*, retinal vasculitis

#### Other ocular adverse events prior to the first intraocular inflammation–related adverse event

Other ocular adverse events reported by the study investigators were selected to examine whether they were reported before the onset of the first IOI-related adverse event and might be a signal of a possible upcoming IOI-related event (Table 4). We first indicate whether these ocular events were reported more frequently in patients with IOI cases than in patients without IOI cases over the duration of the study. Altogether, these ocular events were reported at a higher rate in patients with IOI cases than in patients without IOI cases during the study (58.0% vs 30.4%). Increased intraocular pressure (20.0% vs 3.2%), reduced visual acuity (18.0% vs 7.6%), and retinal hemorrhage (12.0% vs 3.9%) were reported more frequently in patients with IOI cases than in patients without IOI cases. Vitreous floaters (10.0% vs 5.6%), conjunctivitis (8.0% vs 2.2%), blurred vision (8.0% vs 2.5%), and eye pain (8.0% vs 5.3%) were reported at slightly higher rates in patients with IOI cases than in patients without IOI cases. These ocular adverse events were reported before the onset of the first IOI-related adverse event in 28% of patients with IOI cases; however, the incidence of any specific ocular adverse event before the onset of the first IOI-related adverse event was low (≤ 6%).

**Table 4:** Other reported ocular adverse events. The incidence of prespecified treatment-emergent ocular adverse events (AEs) (other than events used to define intraocular inflammation [IOI]–related AEs) in the study eye before the onset of the first IOI-related AE^a^ for patients with definite/probable IOI cases, at any time during the study for patients with definite/probable IOI cases, or at any time during the study for patients without definite/probable IOI cases

Treatment-emergent ocular AE^b,c^	Before the onset of the first IOI-related AE^a^: patients with IOI cases (*N* = 50)	During the study: patients with IOI cases (*N* = 50)	During the study: patients without IOI cases (*N* = 1038)
At least 1 event, *n* (%)	14 (28.0)	29 (58.0)	316 (30.4)
Intraocular pressure increased	3 (6.0)	10 (20.0)	33 (3.2)
Visual acuity reduced	3 (6.0)	9 (18.0)	79 (7.6)
Retinal hemorrhage	2 (4.0)	6 (12.0)	41 (3.9)
Vitreous floaters	1 (2.0)	5 (10.0)	58 (5.6)
Conjunctivitis	3 (6.0)	4 (8.0)	23 (2.2)
Eye pain	3 (6.0)	4 (8.0)	55 (5.3)
Vision blurred	1 (2.0)	4 (8.0)	26 (2.5)
Conjunctival hyperemia	0	2 (4.0)	8 (0.8)
Blindness	1 (2.0)	1 (2.0)	3 (0.3)
Corneal endotheliitis	0	1 (2.0)	0
Episcleritis	0	1 (2.0)	2 (0.2)
Foreign body sensation	1 (2.0)	1 (2.0)	12 (1.2)
Lacrimation increased	0	1 (2.0)	14 (1.3)
Ocular hyperemia	0	1 (2.0)	6 (0.6)
Vitreal cells	1 (2.0)	1 (2.0)	4 (0.4)
Abnormal sensation in eyes	0	0	1 (0.1)
Blindness transient	0	0	0
Corneal edema	0	0	4 (0.4)
Erythema	0	0	2 (0.2)
Keratitis	0	0	3 (0.3)
Noninfective conjunctivitis	0	0	1 (0.1)
Ocular discomfort	0	0	13 (1.3)
Photophobia	0	0	2 (0.2)

^a^IOI-related adverse events were defined as IOI, retinal vascular occlusion, or endophthalmitis

^b^A patient with multiple adverse events is counted only once in each row

^c^Preferred terms based on the *Medical Dictionary for Regulatory Activities* version 20.1 were used for reporting

*AE*, adverse event; *IOI*, intraocular inflammation

## Discussion

Brolucizumab is an anti-VEGF drug first approved in 2019 for the treatment of nAMD that has shown superiority in anatomical outcomes [6, 14, 19]. The post-approval reports of RV or RO typically associated with IOI following treatment with brolucizumab prompted the need to identify early signs and the time course of these events. The present post hoc analysis of the HAWK and HARRIER studies found that in 6 of 12 patients with RV or RO adverse events reported by the study investigators (of the 50 patients with definite/probable IOI cases identified by the SRC), RV or RO was preceded by IOI. This finding is consistent with post-marketing studies that found that signs or symptoms of IOI preceded RV or RO in some patients [25, 26]. The preceding IOI in the present analysis was found to occur in various locations, including the anterior segment. The finding that the location of the preceding IOI can, in some cases, be anterior is consistent with a post-marketing study that found that IOI associated with RV or RO was anterior only, posterior only, both anterior and posterior, or absent [25]. In the other 6 of 12 patients with RV or RO in the present analysis, IOI was not detected before the RV or RO, although it is unknown whether preceding IOI was absent or was not detected by the study investigators. Nevertheless, because IOI has been found to precede RV and RO in some cases, and post-marketing studies have found that IOI typically occurs in association with RV and RO, detection of IOI may prompt additional examination and monitoring for RV and RO [25–29]. Notably, brolucizumab is contraindicated in the presence of active IOI, and it is possible that further injections may exacerbate the IOI.

For almost half of patients in the present analysis, the first IOI-related adverse event was diagnosed in the first 3 months of treatment (≤ 3 months; Fig. 2a); for 26% of patients, the event was diagnosed in months 4 to 6 (> 3 months to ≤ 6 months); and in the remaining 26%, the event was diagnosed after 6 months. This suggests that vigilance for signs of IOI and patient counseling on symptoms of IOI over the first 6 months of treatment may help detect the majority of IOI and RO events. However, clinicians may continue to monitor for signs of IOI and RO after 6 months of treatment in case the onset of the adverse event is delayed. Note that the intervals reported between the first injection of brolucizumab and the first IOI-related adverse events in the present analysis were observed in patients who were anti-VEGF treatment-naïve at the initiation of the studies. It is unknown whether the time course would be different for previously treated patients switched to brolucizumab from a different anti-VEGF. Post-marketing studies of RV or RO have primarily reported events following 1 to 3 intravitreal injections in eyes that were switched to brolucizumab [25, 26, 32]. However, these data are limited in that they do not indicate the frequency of the events as a proportion of those who received the drug. An important consideration for patients switched from another anti-VEGF may be history of IOI or RO. A recent retrospective study of post-marketing data suggests that history of IOI or RO in the 12 months before the initiation of brolucizumab treatment could be a risk factor for IOI-related events with brolucizumab [33]. However, lack of a history of IOI or RO does not imply that patients may not develop an IOI-related event with brolucizumab.

The onset of the first IOI-related adverse event in the present analysis was detected a median of 25.5 days (range = 1–91; mean = 22.9) after the preceding injection of brolucizumab (Fig. 3a). In a post-marketing report of patients developing RV or RO following brolucizumab injection, the mean time to the adverse event presentation was approximately 26 days (range = 3–36) [25]. For 49 of 50 patients in the present analysis, the time between the preceding injection and the onset of the first IOI-related adverse event was ≤ 56 days. It is uncertain from the data what proportion of this time reflects the time to event onset and what proportion reflects the time from event onset to diagnosis. Nevertheless, the time between the preceding injection and detection of the IOI-related adverse event with brolucizumab can be considered relatively long (median duration > 3 weeks) [7, 22]. Because of this, patients may be made aware that symptoms of IOI may arise up to several weeks after the brolucizumab injection and physicians may monitor for adverse events at each visit. The mechanism for the IOI-related events following brolucizumab is unknown; however, the long duration between the preceding injection and time to detection of the IOI-related adverse event has led some to hypothesize the mechanism is that of a delayed hypersensitivity reaction [26, 28]. It has also been suggested that VEGF inhibition may be involved in the IOI-related events [26]. Administration of intravitreal anti-VEGF drugs has been associated with vasoconstriction and decreased ocular perfusion [34, 35].

The present analysis found that certain ocular adverse events (e.g., increased ocular pressure and retinal hemorrhage) were more common among patients with IOI cases than among patients without IOI cases (Table 4). However, these adverse events occurred only at low rates before the first IOI-related adverse events, providing no evidence that these events could be used as signals that the eye is at risk of an IOI-related adverse event with brolucizumab. Symptoms that are commonly associated with IOI, such as blurred vision, floaters, and eye pain, were reported at a low rate in the 50 patients with IOI cases in the present analysis. This contrasts with post-marketing studies of RV or RO with brolucizumab, which have found that most patients have at least 1 symptom [25, 26]. The difference between this study and the post-marketing studies in rates of reported symptoms may possibly be because of a difference in the extent to which the physicians encouraged patients to report symptoms.

The HAWK and HARRIER data provide a time course of IOI and RV or RO adverse events in a group of treatment-naïve patients who received brolucizumab over a relatively long period of time, and ocular adverse events could be readily compared between patients who had IOI cases and those who did not. A limitation of the present analysis is the minimal characterization of the adverse events provided in the study records. Few symptoms were reported for the patients with IOI cases. Furthermore, the location of the RV or RO was not available in the data set. The present analysis also relied on the report of the study investigators to describe the time course of the adverse events; however, not every adverse event of RV or RO was detected by the study investigators, as determined by the SRC review [6, 13]. Furthermore, not every brolucizumab-treated eye was reviewed by the SRC. Because there was underdetection of RV by the study investigators, it is difficult to be sure that all the RO adverse events were related to brolucizumab-associated RV as opposed to a systemic condition, such as cardiovascular disease [36]. The present analysis was limited to the 50 patients with definite/probable IOI cases related to brolucizumab as determined by the SRC; however, for the cases reviewed, the SRC always erred on the side of assuming IOI was associated with brolucizumab [20]. Therefore, it is not known whether all RO adverse events in the present analysis were related to brolucizumab. It is also not known whether all IOI adverse events preceding RV or RO were detected by the study investigators. Increased awareness of RV and RO events with brolucizumab since the HAWK and HARRIER studies may increase detection of the spectrum of IOI, RV, and RO events.

### Summary of practices to identify and manage intraocular inflammation, retinal vasculitis, and retinal vascular occlusion

**Fig. 4:**
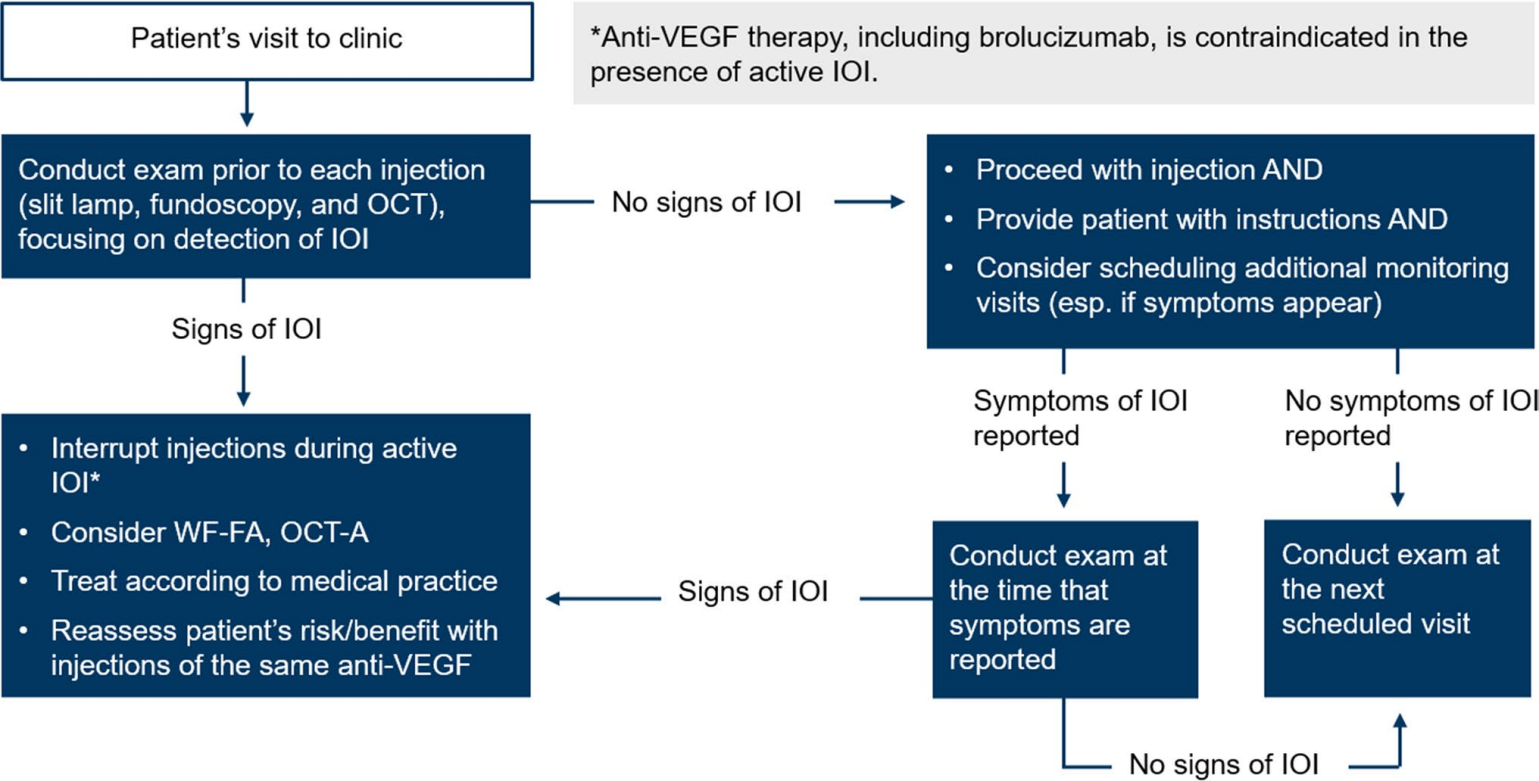
Summary of clinical procedures that may increase detection of the spectrum of intraocular inflammation, retinal vasculitis, and/or retinal vascular occlusion events with brolucizumab. Anti-VEGF, anti-vascular endothelial growth factor; IOI, intraocular inflammation; OCT-A, optical coherence tomography angiography; RV, retinal vasculitis; RO, retinal vascular occlusion; WF-FA, wide-field fluorescein angiography

Based on the literature review and the post hoc analysis of HAWK and HARRIER, the following is a summary of clinical practices that may increase early detection of IOI, RV, and/or RO adverse events associated with brolucizumab (Fig. 4). Many of the procedures are part of standard clinical care and may be performed consistently when using anti-VEGF agents.

1. Conducting a complete ophthalmic exam, including slit-lamp biomicroscopy, dilated fundoscopy, and OCT at each visit before intraocular injection with the aim of interrupting injections for any patient with any signs of active IOI
  Examination for IOI may include evaluation of the anterior and posterior segments, such as evaluation for the presence and extent of anterior chamber cells, anterior chamber flare, vitreous cells, and vitreous haze or debris [25, 26, 37, 38]. OCT may be helpful in examining for the presence of cells, which may appear as hyperreflective dots in the vitreous or within the retina [39, 40]. Other ocular signs to watch for include keratic precipitates, conjunctival injection, Descemet’s folds, and corneal edema [26, 29]. To detect the presence of IOI affecting the vasculature, the retina may be evaluated for the presence of vascular occlusion, boxcarring, or perivascular sheathing inside and outside the macula [25, 41].
2. Educating patients on the symptoms of IOI and instructing patients to seek care from an ophthalmologist in case any symptoms of IOI arise
  Patients may be educated on possible symptoms of IOI, including changes in vision, floaters, eye pain or discomfort, eye redness, and light sensitivity [25–29]. Education on symptoms should be followed by instructions to patients to immediately seek care from their ophthalmologist if the patient develops any symptoms. Instructions to patients may be reiterated at each clinic visit, and patients may be made aware that symptoms may arise up to several weeks after the injection.
  Patient education is likely to affect the extent to which patients follow physician advice [42]. For example, patient education on treatment administration has been shown to improve patient adherence to treatment [43]. Similarly, appropriate education on relevant symptoms and how to evaluate for those symptoms is likely to improve the extent to which patients contact their provider when they experience the symptoms.
  A variety of strategies may help in educating patients on symptoms of IOI (Table 5). Visual aids may be useful for describing symptoms such as floaters and redness. Specifically, visual aids may be used to illustrate how worsening of floaters may appear as an increase in number or size of floaters [44]. Photographs may be useful for explaining the difference between redness due to possible IOI and redness due to hemorrhage at the site of injection. For some symptoms, physicians may need to provide patients with a method for self-evaluation. For example, physicians may need to explain that floaters can be evaluated by looking at a bright background, such as the sky or a light-colored wall. Light sensitivity may be evaluated by intentionally reflecting on one’s reactions and behaviors toward light, such as whether one prefers to avoid daylight more than usual. Providing patients with an opportunity to self-evaluate in the clinic may allow patients to reflect on what normal is for them and may also provide patients with an opportunity to practice monitoring for symptoms of IOI.
3. Considering other imaging modalities, such as wide-field fluorescein angiography (FA) or FA with peripheral sweeps and OCT-A, to further define the nature and extent of the inflammation and/or occlusion if IOI or RO are detected on clinical examination.
  If IOI is detected, it may be beneficial to characterize the full extent of the IOI and further examine for the presence of RV and/or RO so that appropriate treatment decisions can be made. FA will usually show the extent of RV and/or RO better than clinical examination [41]. Important signs to look for on FA include retinal vascular staining, leakage, filling defects, and optic nerve leakage or hyperfluorescence in the macula and the peripheral retina [25, 27–29, 41]. OCT-A may be considered for examining the extent of nonperfusion in the microvasculature [45].
4. Interrupting intravitreal injections if active IOI is observed, and treating the event according to local medical practice

**Table 5:** Strategies to facilitate educating patients with neovascular age-related macular degeneration on symptoms of intraocular inflammation

Symptom of intraocular inflammation	Strategies for patient education on symptoms of intraocular inflammation
Eye pain/discomfort	– Provide patients with a pain-rating scale of simple facial expressions, illustrating magnitude of pain
Floaters	– Use visuals to explain what floaters are and how worsening of floaters may appear as an increase in number or size of floaters
	– Explain how to check for floaters by looking at a bright- or light-colored area
Light sensitivity	– Encourage patients to ask themselves questions on light sensitivity. For example:
	– Do you find yourself wearing sunglasses more often than before?
	– Do you prefer to be in dark or dim light more often than before?
Redness	– Educate patients on the difference between redness due to possible inflammation and redness due to hemorrhage at the site of injection, ideally using photographs of each type of redness

Anti-VEGF therapy, including brolucizumab, is contraindicated in the presence of active IOI [46–50], and there is the potential that further injections may exacerbate the IOI [28]. In some patients, the IOI may progress to RV or RO [25, 26].

### Conclusion

The present post hoc analysis of the brolucizumab arms of the HAWK and HARRIER studies found that among the 50 patients with definite/probable IOI cases identified by the SRC, 12 patients were diagnosed with RV or RO by the study investigators. Six of the 12 patients showed IOI adverse events before the RV or RO adverse events. Consistent and comprehensive monitoring for IOI and patient counseling on symptoms of IOI may therefore be beneficial, so IOI is detected early and can signal the need for additional examination and monitoring for RV and RO.

## Supplementary Information

Below is the link to the electronic supplementary material.

Supplementary file1 (DOCX 17 KB)

Supplementary file2 (DOCX 18 KB)

Supplementary file3 (DOCX 17 KB)

Supplementary file4 (DOCX 60 KB)

Supplementary file5 (DOCX 18 KB)

Supplementary file6 (DOCX 17 KB)

Supplementary file7 (DOCX 46 KB)

## Data Availability

Data are available in Online Resource 4.
