# Supplementary material for: Manifestations of intraocular inflammation over time in patients on brolucizumab for neovascular AMD
Source: Graefes Arch Clin Exp Ophthalmol. 2021 Dec 21;260(6):1843–56. doi: 10.1007/s00417-021-05518-0 (PMC9061681; doi:10.1007/s00417-021-05518-0)
Supplement: Supplementary file 1 — Supplementary file1 (DOCX 17 KB) [file 417_2021_5518_MOESM1_ESM.docx]

**Online Resource 1**

Manifestations of Intraocular Inflammation Over Time in Patients on Brolucizumab for Neovascular AMD

Graefe’s Archive for Clinical and Experimental Ophthalmology

Ramin Khoramnia^1^; Marta S. Figueroa^2^; Lars-Olof Hattenbach^3^; Carlos E. Pavesio^4^; Majid Anderesi^5^; Robert Schmouder^6^; Yu Chen^6^; Marc D. de Smet^7^

^1^The David J. Apple Center for Vision Research, Department of Ophthalmology, University of Heidelberg, Heidelberg, Germany

^2^Retina Division, Ramón y Cajal University Hospital, Madrid, Spain

^3^Department of Ophthalmology, Ludwigshafen Hospital, Ludwigshafen am Rhein, Germany

^4^Department of Uveitis, Moorfields Eye Hospital and UCL, London, United Kingdom

^5^Novartis Pharma AG, Basel, Switzerland

^6^Novartis Pharmaceuticals Corporation, East Hanover, New Jersey, United States

^7^Medical/Surgical Retina and Ocular Inflammation, Microinvasive Ocular Surgery Center (MIOS sa), Lausanne, Switzerland

**Corresponding Author:** Ramin Khoramnia, International Vision Correction Research Centre, University Eye Clinic Heidelberg Im Neuenheimer Feld 400, 69120 Heidelberg; phone: +49 6221 56-39624; fax: +49 6221 56-8229; email: ramin.khoramnia@med.uni-heidelberg.de

**Appendix**

**Literature search strategy**

The following terms were used for the PubMed literature search to identify relevant articles (published on or before August 28, 2020) on signs and symptoms of intraocular inflammation following anti–vascular endothelial growth factor therapy in neovascular age-related macular degeneration:

(“Retinal vasculitis” OR “Ocular vasculitis” OR “Retinitis” OR “Choroidal infarction” OR “Macular ischaemia” OR “Macular ischemia” OR “Ocular ischaemic syndrome” OR “Ocular ischemic syndrome” OR “Retinal artery occlusion” OR “Retinal artery thrombosis” OR “Retinal artery embolism” OR “Retinal artery stenosis” OR “Retinal infarction” OR “Retinal ischaemia” OR “Retinal ischemia” OR “Retinal vein occlusion” OR “Retinal vein thrombosis” OR “Retinal vascular occlusion” OR “Retinal vascular thrombosis” OR “Blindness” OR “Visual impairment” OR “Visual acuity reduced” OR “Anterior chamber cell” OR “Anterior chamber fibrin” OR “Anterior chamber flare” OR “Anterior chamber inflammation” OR “Chorioretinitis” OR “Choroiditis” OR “Cyclitic membrane” OR “Cyclitis” OR “Eye inflammation” OR “Idiopathic orbital inflammation” OR “Iridocyclitis” OR “Iritis” OR “Keratic precipitates” OR “Ocular pemphigoid” OR “Oculomucocutaneous syndrome” OR “Oculorespiratory syndrome” OR “Optic neuritis” OR “Papillitis” OR “Toxic anterior segment syndrome” OR “Tubulointerstitial nephritis” OR “Uveitis” OR “Vitreous haze” OR “Vitritis” OR “Endophthalmitis” OR “Panophthalmitis” OR “Pseudoendophthalmitis” OR “Birdshot chorioretinopathy” OR “Cholesterolosis bulbi” OR “Cogan’s syndrome” OR “Eye infection” OR “Intraocular infection” OR “Hypopyon” OR “Ocular sarcoidosis” OR “Viral keratouveitis” OR “Vitreous abscess” OR “Vogt-Koyanagi-Harada disease” OR “Retinal vasculitis” OR “Retinal artery branch occlusion” OR “Central retinal vein occlusion” OR “Branch retinal vein occlusion” OR “Ischemic optic neuropathy” OR “Posterior synechiae” OR “chorioretinal inflammation” OR “Exudative retinopathy” OR “Optic neuritis” OR “Fuchs” OR “VKH” OR “Vogt-Koyanagi-Harada” OR “Harada disease” OR “Vitreous haemorrhage” OR “Vitreous cell” OR “Vitreous haze” OR “Vitreous flare” OR “RAO” OR “RVO” OR “Intraocular inflammation” OR “IOI” OR “Intra-ocular inflammation” OR “Eye stroke” OR “Arterial occlusive disease” OR “Hollenhorst plaque” OR “optic nerve edema” OR “Retinal arteritis” OR “perivenular phlebitis” OR “Kyrieleis plaques” OR “frosted branch angiitis” OR ocular adverse events OR ocular complications OR ocular safety OR ocular AE OR ocular incidence OR ocular report OR ocular observation) AND (“ranibizumab” OR “Lucentis” OR “bevacizumab” OR “avastin” OR “aflibercept” OR “eylea” OR “brolucizumab” OR “beovu” OR “anti-VEGF” OR “anti VEGF” OR “anti-vascular endothelial growth factor” OR “anti vascular endothelial growth factor”) AND (“age related macular degeneration” OR “age-related macular degeneration” OR “nAMD” OR “neovascular AMD” OR “neovascular age related macular degeneration” OR “wAMD” OR “wet AMD” OR “wet age related macular degeneration” OR “exudative AMD” OR “exudative age related macular degeneration”).
