# Supplementary material for: Manifestations of intraocular inflammation over time in patients on brolucizumab for neovascular AMD
Source: Graefes Arch Clin Exp Ophthalmol. 2021 Dec 21;260(6):1843–56. doi: 10.1007/s00417-021-05518-0 (PMC9061681; doi:10.1007/s00417-021-05518-0)
Supplement: Supplementary file 2 — Supplementary file2 (DOCX 18 KB) [file 417_2021_5518_MOESM2_ESM.docx]

**Online Resource 2**

Manifestations of Intraocular Inflammation Over Time in Patients on Brolucizumab for Neovascular AMD

Graefe’s Archive for Clinical and Experimental Ophthalmology

Ramin Khoramnia^1^; Marta S. Figueroa^2^; Lars-Olof Hattenbach^3^; Carlos E. Pavesio^4^; Majid Anderesi^5^; Robert Schmouder^6^; Yu Chen^6^; Marc D. de Smet^7^

^1^The David J. Apple Center for Vision Research, Department of Ophthalmology, University of Heidelberg, Heidelberg, Germany

^2^Retina Division, Ramón y Cajal University Hospital, Madrid, Spain

^3^Department of Ophthalmology, Ludwigshafen Hospital, Ludwigshafen am Rhein, Germany

^4^Department of Uveitis, Moorfields Eye Hospital and UCL, London, United Kingdom

^5^Novartis Pharma AG, Basel, Switzerland

^6^Novartis Pharmaceuticals Corporation, East Hanover, New Jersey, United States

^7^Medical/Surgical Retina and Ocular Inflammation, Microinvasive Ocular Surgery Center (MIOS sa), Lausanne, Switzerland

**Corresponding Author:** Ramin Khoramnia, International Vision Correction Research Centre, University Eye Clinic Heidelberg Im Neuenheimer Feld 400, 69120 Heidelberg; phone: +49 6221 56-39624; fax: +49 6221 56-8229; email: ramin.khoramnia@med.uni-heidelberg.de

**Preferred Terms Used to Define Intraocular Inflammation.** Terms used to define intraocular inflammation in the present post hoc analysis of the HAWK and HARRIER studies.

| **Preferred Term^a^** |
| --- |
| Anterior chamber cell |
| Anterior chamber fibrin |
| Anterior chamber flare |
| Anterior chamber inflammation |
| Aqueous fibrin |
| Chorioretinitis |
| Choroiditis |
| Cogan’s syndrome |
| Cyclitic membrane |
| Cyclitis |
| Eye infection intraocular |
| Eye inflammation |
| Hypopyon |
| Idiopathic orbital inflammation |
| Infective uveitis |
| Iridocyclitis |
| Iritis |
| Keratic precipitates |
| Noninfective chorioretinitis |
| Noninfective retinitis |
| Ocular pemphigoid |
| Ocular vasculitis |
| Oculomucocutaneous syndrome |
| Oculorespiratory syndrome |
| Ophthalmia neonatorum |
| Optic neuritis |
| Retinal perivascular sheathing |
| Retinal vasculitis |
| Retinitis |
| Toxic anterior segment syndrome |
| Tubulointerstitial nephritis and uveitis syndrome |
| Uveitis |
| Uveitis-glaucoma-hyphema syndrome |
| Viral keratouveitis |
| Viral uveitis |
| Vitreous abscess |
| Vitreous haze |
| Vitritis |
| Vogt-Koyanagi-Harada syndrome |

^a^Preferred terms based on *Medical Dictionary for Regulatory Activities* version 20.1.
