# Supplementary material for: Manifestations of intraocular inflammation over time in patients on brolucizumab for neovascular AMD
Source: Graefes Arch Clin Exp Ophthalmol. 2021 Dec 21;260(6):1843–56. doi: 10.1007/s00417-021-05518-0 (PMC9061681; doi:10.1007/s00417-021-05518-0)
Supplement: Supplementary file 3 — Supplementary file3 (DOCX 17 KB) [file 417_2021_5518_MOESM3_ESM.docx]

**Online Resource 3**

Manifestations of Intraocular Inflammation Over Time in Patients on Brolucizumab for Neovascular AMD

Graefe’s Archive for Clinical and Experimental Ophthalmology

Ramin Khoramnia^1^; Marta S. Figueroa^2^; Lars-Olof Hattenbach^3^; Carlos E. Pavesio^4^; Majid Anderesi^5^; Robert Schmouder^6^; Yu Chen^6^; Marc D. de Smet^7^

^1^The David J. Apple Center for Vision Research, Department of Ophthalmology, University of Heidelberg, Heidelberg, Germany

^2^Retina Division, Ramón y Cajal University Hospital, Madrid, Spain

^3^Department of Ophthalmology, Ludwigshafen Hospital, Ludwigshafen am Rhein, Germany

^4^Department of Uveitis, Moorfields Eye Hospital and UCL, London, United Kingdom

^5^Novartis Pharma AG, Basel, Switzerland

^6^Novartis Pharmaceuticals Corporation, East Hanover, New Jersey, United States

^7^Medical/Surgical Retina and Ocular Inflammation, Microinvasive Ocular Surgery Center (MIOS sa), Lausanne, Switzerland

**Corresponding Author:** Ramin Khoramnia, International Vision Correction Research Centre, University Eye Clinic Heidelberg Im Neuenheimer Feld 400, 69120 Heidelberg; phone: +49 6221 56-39624; fax: +49 6221 56-8229; email: ramin.khoramnia@med.uni-heidelberg.de

**Preferred Terms Used to Define Retinal Vascular Occlusion.** Terms used to define retinal vascular occlusion for the present post hoc analysis of the HAWK and HARRIER studies.

| **Preferred Term^a^** |
| --- |
| Macular ischemia |
| Ocular ischemic syndrome |
| Retinal artery embolism |
| Retinal artery occlusion |
| Retinal artery stenosis |
| Retinal artery thrombosis |
| Retinal infarction |
| Retinal ischemia |
| Retinal vascular occlusion |
| Retinal vascular thrombosis |

^a^Preferred terms based on *Medical Dictionary for Regulatory Activities* version 20.1.
