# Supplementary material for: Manifestations of intraocular inflammation over time in patients on brolucizumab for neovascular AMD
Source: Graefes Arch Clin Exp Ophthalmol. 2021 Dec 21;260(6):1843–56. doi: 10.1007/s00417-021-05518-0 (PMC9061681; doi:10.1007/s00417-021-05518-0)
Supplement: Supplementary file 4 — Supplementary file4 (DOCX 60 KB) [file 417_2021_5518_MOESM4_ESM.docx]

**Online Resource 4**

Manifestations of Intraocular Inflammation Over Time in Patients on Brolucizumab for Neovascular AMD

Graefe’s Archive for Clinical and Experimental Ophthalmology

Ramin Khoramnia^1^; Marta S. Figueroa^2^; Lars-Olof Hattenbach^3^; Carlos E. Pavesio^4^; Majid Anderesi^5^; Robert Schmouder^6^; Yu Chen^6^; Marc D. de Smet^7^

^1^The David J. Apple Center for Vision Research, Department of Ophthalmology, University of Heidelberg, Heidelberg, Germany

^2^Retina Division, Ramón y Cajal University Hospital, Madrid, Spain

^3^Department of Ophthalmology, Ludwigshafen Hospital, Ludwigshafen am Rhein, Germany

^4^Department of Uveitis, Moorfields Eye Hospital and UCL, London, United Kingdom

^5^Novartis Pharma AG, Basel, Switzerland

^6^Novartis Pharmaceuticals Corporation, East Hanover, New Jersey, United States

^7^Medical/Surgical Retina and Ocular Inflammation, Microinvasive Ocular Surgery Center (MIOS sa), Lausanne, Switzerland

**Corresponding Author:** Ramin Khoramnia, International Vision Correction Research Centre, University Eye Clinic Heidelberg Im Neuenheimer Feld 400, 69120 Heidelberg; phone: +49 6221 56-39624; fax: +49 6221 56-8229; email: ramin.khoramnia@med.uni-heidelberg.de

**Ocular Adverse Events in Patients With Definite/Probable Intraocular Inflammation Cases (N=50).** Ocular adverse events in the study eye reported by the study investigators of HAWK and HARRIER for the brolucizumab-treated patients who developed definite/probable intraocular inflammation cases according to the opinion of the independent safety review committee.

| **Subject** | **Sex** | **Serious Event** | **Severity^a^** | **Adverse Event (Preferred Term)^b^** | **Number of Injections^c^** | **Days Since First Day of Study Treatment** | **Days Since Preceding Injection** | **Duration (Days)** | **Action Taken^d^** |
| --- | --- | --- | --- | --- | --- | --- | --- | --- | --- |
| 1 | F | N | 1 | Endophthalmitis | 2 | 38 | 8 | 167 | 3 |
|  |  | N | 1 | Intraocular pressure increased | 2 | 101 | 71 | 20 | 4 |
|  |  | N | 2 | Uveitis | 2 | 101 | 71 | 6 | 4 |
| 2 | F | N | 1 | Keratic precipitates | 4 | 173 | 32 | 14 | 2 |
|  |  | N | 1 | Keratic precipitates | 5 | 233 | 28 | 29 | 4 |
| 3 | M | N | 1 | Conjunctival hemorrhage | 3 | 115 | 58 | 15 | 1 |
|  |  | N | 1 | Eye pain | 3 | 115 | 58 | 1 | 1 |
|  |  | N | 1 | Foreign body sensation in eyes | 3 | 115 | 58 | 15 | 1 |
|  |  | Y | 2 | Endophthalmitis | 4 | 120 | 5 | 23 | 1 |
|  |  | N | 2 | Dry eye | 5 | 168 | 3 | Ongoing | 1 |
|  |  | N | 1 | Blepharitis | 5 | 169 | 4 | 29 | 1 |
|  |  | N | 1 | Conjunctival hemorrhage | 8 | 395 | 51 | 22 | 1 |
|  |  | N | 1 | Vitreous floaters | 12 | 619 | 63 | 20 | 1 |
| 4 | M | N | 2 | Iridocyclitis | 3 | 77 | 20 | 16 | 1 |
|  |  | N | 3 | Iridocyclitis | 3 | 113 | 56 | 135 | 1 |
|  |  | N | 3 | Visual acuity reduced | 3 | 113 | 56 | 7 | 2 |
|  |  | Y | 3 | Retinal artery occlusion | 4 | 119 | 6 | 1 | 3 |
|  |  | N | 2 | Intraocular pressure increased | 4 | 164 | 51 | 34 | 4 |
|  |  | Y | 2 | Uveitis | 4 | 164 | 51 | 202 | 4 |
|  |  | N | 2 | Conjunctival hyperemia | 4 | 197 | 84 | 169 | 4 |
|  |  | N | 2 | Episcleritis | 4 | 488 | 375 | 46 | 4 |
|  |  | N | 1 | Uveitis | 4 | 603 | 490 | 36 | 4 |
| 5 | M | N | 3 | Conjunctival hemorrhage | 1 | 3 | 2 | 27 | 1 |
|  |  | N | 1 | Retinal artery occlusion | 2 | 57 | 28 | Ongoing | 2 |
|  |  | N | 1 | Vitreous floaters | 2 | 88 | 59 | 82 | 1 |
|  |  | N | 1 | Dry age-related macular degeneration | 4 | 281 | 49 | Ongoing | 1 |
|  |  | N | 1 | Vision blurred | 5 | 323 | 14 | 43 | 1 |
| 6 | F | N | 2 | Chorioretinitis | 4 | 159 | 11 | 22 | 1 |
|  |  | N | 1 | Vitreous floaters | 4 | 190 | 42 | 4 | 1 |
|  |  | N | 1 | Intraocular pressure increased | 4 | 193 | 45 | 12 | 1 |
|  |  | N | 1 | Vitreous floaters | 4 | 193 | 45 | 12 | 1 |
|  |  | N | 2 | Vitreous floaters | 4 | 197 | 49 | 8 | 1 |
|  |  | N | 2 | Vitreous floaters | 4 | 204 | 56 | 25 | 2 |
|  |  | Y | 2 | Retinopathy proliferative | 4 | 228 | 80 | 8 | 3 |
| 7 | M | N | 1 | Retinal artery occlusion | 8 | 371 | 35 | 44 | 1 |
|  |  | Y | 3 | Retinal artery occlusion | 8 | 414 | 78 | 8 | 1 |
| 8 | F | N | 2 | Endophthalmitis | 7 | 369 | 3 | 27 | 1 |
| 9 | F | N | 2 | Retinal artery occlusion | 3 | 109 | 49 | Ongoing | 1 |
|  |  | N | 2 | Corneal endotheliitis | 4 | 201 | 57 | 11 | 1 |
|  |  | N | 2 | Iritis | 4 | 201 | 57 | 11 | 1 |
|  |  | N | 1 | Corneal endotheliitis | 5 | 307 | 76 | 30 | 1 |
|  |  | N | 1 | Corneal endotheliitis | 6 | 362 | 55 | 65 | 1 |
|  |  | N | 1 | Retinal hemorrhage | 6 | 362 | 55 | 35 | 1 |
|  |  | N | 1 | Meibomianitis | 6 | 396 | 89 | 88 | 1 |
|  |  | N | 2 | Iritis | 8 | 474 | 29 | 10 | 1 |
|  |  | N | 2 | Corneal endotheliitis | 9 | 557 | 52 | 17 | 2 |
|  |  | N | 2 | Iritis | 9 | 557 | 52 | 17 | 2 |
|  |  | N | 1 | Retinal hemorrhage | 9 | 585 | 80 | 36 | 1 |
|  |  | N | 2 | Corneal endotheliitis | 9 | 620 | 115 | Ongoing | 2 |
|  |  | N | 2 | Vitritis | 9 | 620 | 115 | 16 | 2 |
|  |  | N | 1 | Corneal endotheliitis | 9 | 683 | 178 | Ongoing | 4 |
|  |  | N | 1 | Uveitic glaucoma | 9 | 683 | 178 | Ongoing | 4 |
| 10 | M | N | 2 | Intraocular pressure increased | 1 | 15 | 14 | 51 | 1 |
|  |  | N | 1 | Anterior chamber inflammation | 1 | 17 | 16 | 21 | 1 |
| 11 | F | N | 1 | Dry eye | 2 | 57 | 31 | 5 | 1 |
|  |  | N | 2 | Retinal vasculitis | 2 | 57 | 31 | 278 | 2 |
|  |  | N | 2 | Cataract | 7 | 446 | 21 | 170 | 1 |
| 12 | M | N | 1 | Vitritis | 3 | 62 | 8 | 79 | 1 |
|  |  | N | 1 | Iritis | 5 | 228 | 4 | 81 | 2 |
| 13 | M | N | 2 | Uveitis | 2 | 58 | 31 | 280 | 2 |
|  |  | N | 1 | Blepharitis | 4 | 298 | 157 | 103 | 1 |
|  |  | Y | 2 | Endophthalmitis | 4 | 337 | 196 | 176 | 3 |
|  |  | N | 1 | Glaucoma | 5 | 365 | 28 | 148 | 3 |
| 14 | M | N | 1 | Iritis | 1 | 29 | 28 | 29 | 1 |
|  |  | N | 1 | Retinal Tear | 4 | 197 | 49 | Ongoing | 1 |
| 15 | F | N | 3 | Blindness | 5 | 272 | 49 | Ongoing | 1 |
|  |  | N | 3 | Retinal hemorrhage | 5 | 272 | 49 | Ongoing | 1 |
|  |  | Y | 3 | Endophthalmitis | 7 | 339 | 4 | 27 | 1 |
|  |  | N | 1 | Vision blurred | 7 | 370 | 35 | Ongoing | 1 |
|  |  | N | 1 | Vitreous hemorrhage | 7 | 386 | 51 | 99 | 4 |
|  |  | N | 1 | Eye pain | 7 | 477 | 142 | 10 | 4 |
|  |  | N | 1 | Vitreous hemorrhage | 7 | 512 | 177 | 57 | 4 |
| 16 | F | N | 1 | Vitreous detachment | 2 | 57 | 28 | Ongoing | 1 |
|  |  | N | 1 | Chorioretinitis | 3 | 60 | 3 | 105 | 1 |
|  |  | N | 1 | Conjunctival hemorrhage | 3 | 143 | 86 | 5 | 1 |
|  |  | N | 1 | Uveitis | 4 | 164 | 21 | 41 | 2 |
| 17 | F | N | 1 | Eye pruritus | 0 | -13 | NA | 2 | 4 |
|  |  | N | 2 | Retinal hemorrhage | 3 | 106 | 40 | 38 | 1 |
|  |  | N | 1 | Blister | 4 | 148 | 42 | 8 | 1 |
|  |  | N | 2 | Iritis | 9 | 451 | 44 | 20 | 1 |
|  |  | N | 1 | Conjunctival hyperemia | 12 | 594 | 31 | 82 | 1 |
| 18 | F | N | 1 | Blepharitis | 5 | 259 | 35 | 31 | 1 |
|  |  | N | 2 | Conjunctival hemorrhage | 5 | 320 | 96 | 40 | 1 |
|  |  | N | 1 | Iridocyclitis | 8 | 492 | 2 | 125 | 1 |
| 19 | F | N | 1 | Eye inflammation | 3 | 60 | 3 | Ongoing | 3 |
|  |  | N | 1 | Visual acuity reduced | 3 | 90 | 33 | Ongoing | 4 |
| 20 | F | N | 1 | Retinal tear | 3 | 69 | 12 | 3 | 1 |
|  |  | N | 1 | Conjunctivitis | 3 | 78 | 21 | 18 | 1 |
|  |  | Y | 3 | Uveitis | 4 | 182 | 41 | 65 | 3 |
| 21 | F | N | 2 | Vitritis | 3 | 85 | 28 | 39 | 1 |
|  |  | N | 1 | Retinal tear | 3 | 123 | 66 | 1 | 1 |
|  |  | N | 1 | Cataract nuclear | 3 | 148 | 91 | 123 | 1 |
|  |  | N | 1 | Visual acuity reduced | 6 | 344 | 42 | 71 | 1 |
|  |  | N | 1 | Visual acuity reduced | 9 | 589 | 14 | Ongoing | 1 |
| 22 | F | N | 2 | Anterior chamber inflammation | 2 | 29 | 1 | 18 | 1 |
|  |  | N | 1 | Vitritis | 4 | 122 | 10 | 13 | 1 |
| 23 | F | N | 1 | Vision blurred | 3 | 140 | 83 | 29 | 1 |
|  |  | N | 1 | Eye pain | 4 | 197 | 57 | 4 | 1 |
|  |  | N | 2 | Uveitis | 5 | 249 | 25 | 12 | 1 |
| 24 | F | N | 1 | Iritis | 1 | 32 | 31 | 15 | 1 |
|  |  | N | 2 | Retinal artery embolism | 4 | 202 | 57 | Ongoing | 1 |
| 25 | M | N | 1 | Intraocular pressure increased | 2 | 59 | 29 | 3 | 1 |
|  |  | Y | 1 | Uveitis | 3 | 85 | 26 | Ongoing | 3 |
| 26 | F | N | 2 | Intraocular pressure increased | 5 | 246 | 28 | Ongoing | 1 |
|  |  | N | 2 | Uveitis | 5 | 246 | 28 | Ongoing | 2 |
| 27 | F | N | 1 | Vitreous floaters | 3 | 78 | 21 | 15 | 1 |
|  |  | N | 1 | Anterior chamber cell | 3 | 92 | 35 | 19 | 1 |
|  |  | N | 1 | Punctate keratitis | 3 | 92 | 35 | 19 | 1 |
|  |  | N | 2 | Iritis | 3 | 100 | 43 | 11 | 1 |
| 28 | F | N | 2 | Ocular hypertension | 8 | 460 | 36 | Ongoing | 1 |
|  |  | N | 2 | Uveitis | 8 | 460 | 36 | 49 | 2 |
|  |  | N | 2 | Uveitis | 9 | 560 | 24 | Ongoing | 3 |
| 29 | F | N | 2 | Uveitis | 1 | 18 | 17 | Ongoing | 1 |
|  |  | N | 1 | Vitreous floaters | 1 | 18 | 17 | 233 | 1 |
|  |  | Y | 3 | Retinal artery thrombosis | 1 | 33 | 32 | Ongoing | 3 |
|  |  | N | 1 | Retinal hemorrhage | 1 | 47 | 46 | 204 | 4 |
| 30 | F | N | 1 | Iridocyclitis | 3 | 148 | 91 | 8 | 1 |
| 31 | F | N | 2 | Cataract | 5 | 204 | 42 | 279 | 1 |
|  |  | N | 2 | Visual acuity reduced | 5 | 204 | 42 | Ongoing | 1 |
|  |  | N | 1 | Arteriosclerotic retinopathy | 5 | 223 | 61 | 260 | 1 |
|  |  | N | 2 | Uveitis | 6 | 252 | 29 | 120 | 2 |
|  |  | N | 1 | Retinal hemorrhage | 8 | 399 | 53 | 57 | 1 |
|  |  | N | 2 | Neovascular age-related macular degeneration | 9 | 427 | 28 | Ongoing | 1 |
|  |  | N | 2 | Macular scar | 12 | 597 | 35 | Ongoing | 1 |
| 32 | F | N | 1 | Keratitis bacterial | 3 | 66 | 8 | 42 | 1 |
|  |  | N | 2 | Vitritis | 3 | 100 | 42 | 36 | 1 |
|  |  | Y | 2 | Vitritis | 3 | 135 | 77 | 56 | 3 |
|  |  | N | 1 | Retinal artery embolism | 3 | 652 | 594 | Ongoing | 4 |
| 33 | F | N | 1 | Keratic precipitates | 3 | 80 | 23 | 69 | 1 |
|  |  | N | 1 | Age-related macular degeneration | 3 | 113 | 56 | Ongoing | 1 |
|  |  | N | 2 | Visual acuity reduced | 3 | 113 | 56 | Ongoing | 1 |
| 34 | M | N | 1 | Retinal perivascular sheathing | 4 | 167 | 28 | Ongoing | 3 |
| 35 | F | N | 2 | Age-related macular degeneration | 4 | 159 | 18 | Ongoing | 1 |
|  |  | N | 2 | Uveitis | 6 | 293 | 19 | 66 | 2 |
|  |  | N | 1 | Retinal hemorrhage | 8 | 421 | 28 | 29 | 1 |
|  |  | N | 1 | Anterior chamber inflammation | 9 | 477 | 28 | 106 | 2 |
|  |  | N | 1 | Intraocular pressure increased | 9 | 477 | 28 | 106 | 2 |
| 36 | F | N | 2 | Uveitis | 2 | 51 | 23 | 287 | 3 |
|  |  | N | 1 | Intraocular pressure increased | 4 | 309 | 189 | 15 | 4 |
|  |  | N | 1 | Blepharitis | 4 | 442 | 322 | 18 | 4 |
| 37 | F | N | 1 | Conjunctivitis | 1 | 12 | 11 | 11 | 1 |
|  |  | N | 2 | Iritis | 2 | 50 | 21 | 183 | 3 |
|  |  | Y | 2 | Cataract | 3 | 120 | 63 | 92 | 4 |
|  |  | N | 1 | Visual acuity reduced | 3 | 120 | 63 | 218 | 4 |
|  |  | N | 1 | Ocular hyperemia | 3 | 247 | 190 | 112 | 4 |
|  |  | N | 1 | Trichiasis | 3 | 247 | 190 | 1 | 4 |
|  |  | N | 1 | Dry eye | 3 | 449 | 392 | Ongoing | 4 |
|  |  | N | 1 | Visual acuity reduced | 3 | 582 | 525 | 36 | 4 |
| 38 | F | N | 1 | Vitreous detachment | 6 | 390 | 82 | Ongoing | 1 |
|  |  | N | 1 | Iritis | 8 | 481 | 5 | 25 | 1 |
|  |  | N | 1 | Iritis | 9 | 556 | 1 | 14 | 1 |
|  |  | N | 1 | Iritis | 10 | 652 | 1 | 22 | 4 |
| 39 | M | N | 2 | Iritis | 1 | 14 | 13 | 108 | 3 |
| 40 | F | N | 1 | Iritis | 4 | 145 | 4 | 369 | 1 |
|  |  | N | 1 | Diabetic retinopathy | 6 | 305 | 3 | 369 | 1 |
| 41 | M | N | 1 | Uveitis | 1 | 12 | 11 | 214 | 1 |
|  |  | N | 1 | Retinal artery occlusion | 2 | 47 | 17 | 607 | 1 |
|  |  | N | 1 | Cataract subcapsular | 5 | 278 | 53 | Ongoing | 1 |
| 42 | F | N | 1 | Iridocyclitis | 1 | 28 | 27 | 66 | 1 |
|  |  | Y | 3 | Retinal artery thrombosis | 2 | 49 | 21 | Ongoing | 3 |
|  |  | Y | 3 | Visual acuity reduced | 2 | 156 | 128 | Ongoing | 4 |
| 43 | F | Y | 3 | Anterior chamber inflammation | 3 | 65 | 8 | 617 | 3 |
|  |  | Y | 3 | Retinal artery embolism | 3 | 65 | 8 | Ongoing | 3 |
|  |  | N | 2 | Blepharitis | 3 | 214 | 157 | 286 | 4 |
|  |  | N | 2 | Conjunctivitis | 3 | 214 | 157 | 468 | 4 |
|  |  | N | 2 | Eye pruritus | 3 | 214 | 157 | 103 | 4 |
|  |  | N | 2 | Choroidal neovascularization | 3 | 499 | 442 | Ongoing | 4 |
| 44 | F | N | 1 | Retinal pigment epithelial tear | 1 | 15 | 14 | Ongoing | 1 |
|  |  | N | 1 | Chorioretinitis | 2 | 56 | 28 | 57 | 2 |
|  |  | N | 1 | Intraocular pressure increased | 2 | 112 | 84 | 14 | 1 |
|  |  | Y | 2 | Uveitis | 3 | 116 | 4 | 117 | 3 |
| 45 | F | N | 1 | Visual acuity reduced | 4 | 173 | 63 | Ongoing | 1 |
|  |  | N | 1 | Anterior chamber flare | 5 | 180 | 7 | 11 | 1 |
|  |  | N | 1 | Vision blurred | 5 | 232 | 59 | 11 | 1 |
|  |  | N | 1 | Blepharitis | 8 | 386 | 52 | Ongoing | 1 |
|  |  | N | 1 | Lacrimation increased | 10 | 629 | 183 | Ongoing | 4 |
| 46 | F | N | 2 | Intraocular pressure increased | 3 | 138 | 83 | 4 | 1 |
|  |  | N | 2 | Intraocular pressure increased | 4 | 147 | 6 | 2 | 1 |
|  |  | Y | 3 | Uveitis | 4 | 156 | 15 | 130 | 3 |
|  |  | N | 1 | Intraocular pressure increased | 4 | 222 | 81 | 9 | 4 |
|  |  | N | 2 | Retinal depigmentation | 4 | 314 | 173 | Ongoing | 4 |
|  |  | N | 1 | Intraocular pressure increased | 4 | 446 | 305 | 4 | 4 |
| 47 | F | N | 1 | Eye inflammation | 5 | 260 | 28 | 25 | 1 |
|  |  | N | 2 | Neovascular age-related macular degeneration | 5 | 316 | 84 | 29 | 1 |
| 48 | M | N | 1 | Vitreal cells | 1 | 29 | 28 | 92 | 1 |
|  |  | N | 2 | Conjunctivitis | 3 | 154 | 97 | 12 | 1 |
|  |  | N | 2 | Eye pain | 4 | 232 | 67 | 2 | 1 |
|  |  | N | 1 | Iridocyclitis | 5 | 260 | 28 | 71 | 1 |
|  |  | N | 1 | Vitritis | 5 | 309 | 77 | 22 | 1 |
|  |  | N | 1 | Anterior chamber flare | 6 | 393 | 84 | 29 | 1 |
|  |  | N | 1 | Anterior chamber flare | 7 | 456 | 63 | 56 | 1 |
|  |  | N | 1 | Conjunctivitis | 8 | 495 | 25 | 13 | 1 |
|  |  | N | 2 | Posterior capsule opacification | 8 | 526 | 56 | Ongoing | 1 |
| 49 | M | N | 1 | Anterior chamber cell | 1 | 29 | 28 | 29 | 1 |
| 50 | F | N | 1 | Visual acuity reduced | 3 | 140 | 85 | 37 | 1 |
|  |  | Y | 2 | Uveitis | 4 | 176 | 36 | 204 | 3 |

^a^Severity: 1 = mild; 2 = moderate; 3 = severe. *Mild* was defined as usually transient in nature and generally not interfering with normal activities. *Moderate* was defined as sufficiently discomforting to interfere with normal activities. *Severe* was defined as preventing normal activities.

^b^*Medical Dictionary for Regulatory Activities* version 20.1 has been used for reporting.

^c^The number of injections is the total number of injections before the adverse event start date.

^d^Action taken with the study treatment: 1 = did not change; 2 = drug interrupted; 3 = drug withdrawn; 4 = not applicable.
