# Supplementary material for: Manifestations of intraocular inflammation over time in patients on brolucizumab for neovascular AMD
Source: Graefes Arch Clin Exp Ophthalmol. 2021 Dec 21;260(6):1843–56. doi: 10.1007/s00417-021-05518-0 (PMC9061681; doi:10.1007/s00417-021-05518-0)
Supplement: Supplementary file 5 — Supplementary file5 (DOCX 18 KB) [file 417_2021_5518_MOESM5_ESM.docx]

**Online Resource 5**

Manifestations of Intraocular Inflammation Over Time in Patients on Brolucizumab for Neovascular AMD

Graefe’s Archive for Clinical and Experimental Ophthalmology

Ramin Khoramnia^1^; Marta S. Figueroa^2^; Lars-Olof Hattenbach^3^; Carlos E. Pavesio^4^; Majid Anderesi^5^; Robert Schmouder^6^; Yu Chen^6^; Marc D. de Smet^7^

^1^The David J. Apple Center for Vision Research, Department of Ophthalmology, University of Heidelberg, Heidelberg, Germany

^2^Retina Division, Ramón y Cajal University Hospital, Madrid, Spain

^3^Department of Ophthalmology, Ludwigshafen Hospital, Ludwigshafen am Rhein, Germany

^4^Department of Uveitis, Moorfields Eye Hospital and UCL, London, United Kingdom

^5^Novartis Pharma AG, Basel, Switzerland

^6^Novartis Pharmaceuticals Corporation, East Hanover, New Jersey, United States

^7^Medical/Surgical Retina and Ocular Inflammation, Microinvasive Ocular Surgery Center (MIOS sa), Lausanne, Switzerland

**Corresponding Author:** Ramin Khoramnia, International Vision Correction Research Centre, University Eye Clinic Heidelberg Im Neuenheimer Feld 400, 69120 Heidelberg; phone: +49 6221 56-39624; fax: +49 6221 56-8229; email: ramin.khoramnia@med.uni-heidelberg.de

**The Number of Days Between the Onset of the First Intraocular Inflammation–Related Adverse Event and Discontinuation From Study Treatment.** The interval (days) between the onset of the first intraocular inflammation (IOI)–related adverse event (AE) and discontinuation from the study treatment (brolucizumab 3 mg or 6 mg) in the subgroup of patients who had a definite/probable IOI case (according to the safety review committee) and discontinued the study treatment.^a,b^

|  | Patients who discontinued treatment without further IOI-related AEs after the onset date of the first IOI-related AE | Patients who discontinued treatment with ≥1 IOI-related AE after the onset date of the first IOI-related AE |
| --- | --- | --- |
| Patients with at least 1 injection after the onset of the first IOI-related AE |  |  |
| n | 2 | 7 |
| Mean (SD) | 67.0 (33.9) | 184.9 (137.0) |
| Median | 67 | 105 |
| Min, Max | 43, 91 | 61, 354 |
| Q1, Q3 | 43, 91 | 65, 343 |
| Patients with no injections after the onset of the first IOI-related AE |  |  |
| n | 12 | 4 |
| Mean (SD) | 30.4 (28.0) | 33.8 (10.5) |
| Median | 27.5 | 33 |
| Min, Max | 1, 94 | 22, 47 |
| Q1, Q3 | 8, 40 | 26, 41.5 |

^a^A total of 26 patients discontinued the study treatment among the 50 patients with an IOI case according to the safety review committee.

^b^One patient (not included in the table) discontinued the study treatment due to lack of efficacy 302 days after a single IOI-related AE, with 5 injections after the onset of the IOI-related AE.

AE = adverse event; IOI = intraocular inflammation.
