# Supplementary material for: Manifestations of intraocular inflammation over time in patients on brolucizumab for neovascular AMD
Source: Graefes Arch Clin Exp Ophthalmol. 2021 Dec 21;260(6):1843–56. doi: 10.1007/s00417-021-05518-0 (PMC9061681; doi:10.1007/s00417-021-05518-0)
Supplement: Supplementary file 6 — Supplementary file6 (DOCX 17 KB) [file 417_2021_5518_MOESM6_ESM.docx]

**Online Resource 6**

Manifestations of Intraocular Inflammation Over Time in Patients on Brolucizumab for Neovascular AMD

Graefe’s Archive for Clinical and Experimental Ophthalmology

Ramin Khoramnia^1^; Marta S. Figueroa^2^; Lars-Olof Hattenbach^3^; Carlos E. Pavesio^4^; Majid Anderesi^5^; Robert Schmouder^6^; Yu Chen^6^; Marc D. de Smet^7^

^1^The David J. Apple Center for Vision Research, Department of Ophthalmology, University of Heidelberg, Heidelberg, Germany

^2^Retina Division, Ramón y Cajal University Hospital, Madrid, Spain

^3^Department of Ophthalmology, Ludwigshafen Hospital, Ludwigshafen am Rhein, Germany

^4^Department of Uveitis, Moorfields Eye Hospital and UCL, London, United Kingdom

^5^Novartis Pharma AG, Basel, Switzerland

^6^Novartis Pharmaceuticals Corporation, East Hanover, New Jersey, United States

^7^Medical/Surgical Retina and Ocular Inflammation, Microinvasive Ocular Surgery Center (MIOS sa), Lausanne, Switzerland

**Corresponding Author:** Ramin Khoramnia, International Vision Correction Research Centre, University Eye Clinic Heidelberg Im Neuenheimer Feld 400, 69120 Heidelberg; phone: +49 6221 56-39624; fax: +49 6221 56-8229; email: ramin.khoramnia@med.uni-heidelberg.de

**Number of Injections After the Onset of the First Intraocular Inflammation–Related Adverse Event in Patients Who Discontinued Study Treatment and Had at Least 1 Injection.** The number of brolucizumab injections received after the onset of the first intraocular inflammation (IOI)-related adverse event (AE) up to discontinuation from the study treatment (brolucizumab 3 mg or 6 mg) in the subgroup of patients who had a definite/probable IOI case (according to the safety review committee), discontinued the study treatment, and had at least 1 injection after the onset of the first IOI-related AE.^a,b^

|  | Patients who discontinued treatment without further IOI-related AEs after the onset date of the first IOI-related AE | Patients who discontinued treatment with ≥1 IOI-related AE after the onset date of the first IOI-related AE |
| --- | --- | --- |
| n | 2 | 7 |
| Mean (SD) | 1.5 (0.7) | 2 (1.5) |
| Median | 1.5 | 1 |
| Min, Max | 1, 2 | 1, 5 |
| Q1, Q3 | 1, 2 | 1, 3 |

^a^A total of 10 patients discontinued the study treatment with at least 1 injection after the onset of the first IOI-related AE among the 50 patients with an IOI case according to the safety review committee.

^b^One patient (not included in the table) discontinued the study treatment due to lack of efficacy 302 days after a single IOI-related AE, with 5 injections after the onset of the IOI-related AE.

AE = adverse event; IOI = intraocular inflammation.
