# Supplementary material for: Manifestations of intraocular inflammation over time in patients on brolucizumab for neovascular AMD
Source: Graefes Arch Clin Exp Ophthalmol. 2021 Dec 21;260(6):1843–56. doi: 10.1007/s00417-021-05518-0 (PMC9061681; doi:10.1007/s00417-021-05518-0)
Supplement: Supplementary file 7 — Supplementary file7 (DOCX 46 KB) [file 417_2021_5518_MOESM7_ESM.docx]

**Online Resource 7**

Manifestations of Intraocular Inflammation Over Time in Patients on Brolucizumab for Neovascular AMD

Graefe’s Archive for Clinical and Experimental Ophthalmology

Ramin Khoramnia^1^; Marta S. Figueroa^2^; Lars-Olof Hattenbach^3^; Carlos E. Pavesio^4^; Majid Anderesi^5^; Robert Schmouder^6^; Yu Chen^6^; Marc D. de Smet^7^

^1^The David J. Apple Center for Vision Research, Department of Ophthalmology, University of Heidelberg, Heidelberg, Germany

^2^Retina Division, Ramón y Cajal University Hospital, Madrid, Spain

^3^Department of Ophthalmology, Ludwigshafen Hospital, Ludwigshafen am Rhein, Germany

^4^Department of Uveitis, Moorfields Eye Hospital and UCL, London, United Kingdom

^5^Novartis Pharma AG, Basel, Switzerland

^6^Novartis Pharmaceuticals Corporation, East Hanover, New Jersey, United States

^7^Medical/Surgical Retina and Ocular Inflammation, Microinvasive Ocular Surgery Center (MIOS sa), Lausanne, Switzerland

**Corresponding Author:** Ramin Khoramnia, International Vision Correction Research Centre, University Eye Clinic Heidelberg Im Neuenheimer Feld 400, 69120 Heidelberg; phone: +49 6221 56-39624; fax: +49 6221 56-8229; email: ramin.khoramnia@med.uni-heidelberg.de

**Concomitant Medication or Nondrug Therapy Given in the Study Eye During the First IOI-Related Adverse Event in Patients With Definite/Probable Intraocular Inflammation Cases (N=50).** Concomitant medication or nondrug therapy during the first for the brolucizumab-treated patients who developed definite/probable intraocular inflammation cases according to the opinion of the independent safety review committee.

| **Subject** | **Serious Event** | **Severity^a^** | **First IOI-Related Adverse Event (Preferred Term)^b^** | **Outcome** | **IOI-Related Adverse Event Onset Day^c^** | **Duration (Days)** | **Concomitant Medication/Therapy (Standardized Term)^d^** | **Concomitant Medication/Therapy Start Day^e^** |
| --- | --- | --- | --- | --- | --- | --- | --- | --- |
| 1 | N | 1 | Endophthalmitis | Resolved | 38 | 167 | Ceftazidime, dexamethasone, prednisolone acetate, vancomycin, moxifloxacin hydrochloride | 20 |
|  |  |  |  |  |  |  | Ranibizumab | 28 |
| 2 | N | 1 | Keratic precipitates | Resolved | 173 | 14 | Loteprednol etabonate | 1 |
|  |  |  |  |  |  |  | Difluprednate | 7 |
|  |  |  |  |  |  |  | Difluprednate | 14 |
| 3 | Y | 2 | Endophthalmitis | Recovered | 120 | 23 | Paracentesis eye, ceftazidime, vancomycin, cefazolin, homatropine, prednisolone acetate, moxifloxacin hydrochloride | 1 |
| 4 | N | 2 | Iridocyclitis | Resolved | 77 | 16 | Prednisolone | 5 |
| 5 | N | 1 | Retinal artery occlusion | Not resolved | 57 | Ongoing | N/A | N/A |
| 6 | N | 2 | Chorioretinitis | Resolved | 159 | 22 | Prednisolone | 1 |
|  |  |  |  |  |  |  | Prednisolone | 11 |
|  |  |  |  |  |  |  | Prednisolone | 15 |
|  |  |  |  |  |  |  | Prednisolone | 19 |
| 7 | N | 1 | Retinal artery occlusion | Resolved | 371 | 44 | N/A | N/A |
| 8 | N | 2 | Endophthalmitis | Resolved with sequelae | 369 | 27 | Eye operation (anterior chamber tap^f^), ultrasound scan, difluprednate, intraocular injection, ofloxacin, eye operation (vitreous tap^f^), ceftazidime, dexamethasone, vancomycin | 1 |
|  |  |  |  |  |  |  | Ultrasound scan | 2 |
|  |  |  |  |  |  |  | Ultrasound scan | 3 |
|  |  |  |  |  |  |  | Difluprednate | 10 |
|  |  |  |  |  |  |  | Difluprednate | 17 |
|  |  |  |  |  |  |  | Difluprednate | 27 |
| 9 | N | 2 | Retinal artery occlusion | Unknown | 109 | Ongoing | N/A | N/A |
| 10 | N | 1 | Anterior chamber inflammation | Resolved | 17 | 21 | Moxifloxacin hydrochloride | 1 |
|  |  |  |  |  |  |  | Brinzolamide | 11 |
| 11 | N | 2 | Retinal vasculitis | Resolved | 57 | 278 | Betamethasone sodium phosphate, moxifloxacin hydrochloride, hyaluronate sodium | 1 |
|  |  |  |  |  |  |  | Hyaluronate sodium | 5 |
| 12 | N | 1 | Vitritis | Resolved with sequelae | 62 | 79 | Moxifloxacin hydrochloride | 2 |
| 13 | N | 2 | Uveitis | Resolved | 58 | 280 | DuoTrav | 7 |
|  |  |  |  |  |  |  | Fluorometholone | 28 |
|  |  |  |  |  |  |  | DuoTrav | 42 |
|  |  |  |  |  |  |  | Fluorometholone, moxifloxacin hydrochloride | 44 |
|  |  |  |  |  |  |  | Betamethasone sodium phosphate, brimonidine tartrate, ripasudil, timolol maleate | 56 |
| 14 | N | 1 | Iritis | Resolved | 29 | 29 | N/A | N/A |
| 15 | Y | 3 | Endophthalmitis | Resolved | 339 | 27 | Atropine, ceftazidime, dexamethasone, difluprednate, vancomycin, moxifloxacin hydrochloride, eye operation (vitreous tap^f^) | 1 |
| 16 | N | 1 | Chorioretinitis | Resolved with sequelae | 60 | 105 | N/A | N/A |
| 17 | N | 2 | Iritis | Resolved | 451 | 20 | Difluprednate | 1 |
| 18 | N | 1 | Iridocyclitis | Resolved | 492 | 125 | Difluprednate, moxifloxacin hydrochloride | 1 |
| 19 | N | 1 | Eye inflammation | Not resolved | 60 | Ongoing | Difluprednate, moxifloxacin hydrochloride | 5 |
| 20 | Y | 3 | Uveitis | Resolved | 182 | 65 | Atropine, dexamethasone sodium phosphate, dorzolamide hydrochloride, moxifloxacin | 4 |
|  |  |  |  |  |  |  | Brimonidine tartrate, intraocular injection (sub-Tenon of betamethasone^f^), timolol | 16 |
| 21 | N | 2 | Vitritis | Resolved | 85 | 39 | Prednisolone acetate | 1 |
|  |  |  |  |  |  |  | Atropine, ciprofloxacin, dexamethasone | 36 |
|  |  |  |  |  |  |  | Dexamethasone, retinal laser coagulation, hyaluronidase, lidocaine, ceftazidime, bupivacaine, vitrectomy, vancomycin | 39 |
| 22 | N | 2 | Anterior chamber inflammation | Resolved | 29 | 18 | Prednisolone acetate | 1 |
| 23 | N | 2 | Uveitis | Resolved | 249 | 12 | Prednisolone acetate | 1 |
| 24 | N | 1 | Iritis | Resolved | 32 | 15 | Prednisolone acetate | 1 |
| 25 | Y | 1 | Uveitis | Not resolved | 85 | Ongoing | Combigan, difluprednate, Simbrinza | 1 |
|  |  |  |  |  |  |  | Prednisolone | 5 |
| 26 | N | 2 | Uveitis | Not resolved | 246 | Ongoing | Prednisolone acetate | 39 |
|  |  |  |  |  |  |  | Combigan, bimatoprost | 40 |
| 27 | N | 1 | Anterior chamber cell | Resolved | 92 | 19 | Atropine, difluprednate | 9 |
| 28 | N | 2 | Uveitis | Resolved | 460 | 49 | Dorzolamide | 4 |
|  |  |  |  |  |  |  | Homatropine, prednisolone acetate | 5 |
|  |  |  |  |  |  |  | Combigan | 14 |
| 29 | N | 2 | Uveitis | Not resolved | 18 | Ongoing | Dexamethasone, prednisolone acetate | 1 |
| 30 | N | 1 | Iridocyclitis | Resolved | 148 | 8 | Prednisolone | 1 |
| 31 | N | 2 | Uveitis | Resolved | 252 | 120 | Prednisolone acetate | 1 |
|  |  |  |  |  |  |  | Prednisolone | 30 |
| 32 | N | 2 | Vitritis | Resolved with sequelae | 100 | 36 | Prednisolone acetate | 2 |
|  |  |  |  |  |  |  | Brimonidine tartrate, prednisolone acetate | 7 |
| 33 | N | 1 | Keratic precipitates | Resolved | 80 | 69 | N/A | N/A |
| 34 | N | 1 | Retinal perivascular sheathing | Not resolved | 167 | Ongoing | N/A | N/A |
| 35 | N | 2 | Uveitis | Resolved | 293 | 66 | DuoTrav | 1 |
|  |  |  |  |  |  |  | Betamethasone sodium phosphate, Mydrin P, prednisolone acetate | 10 |
|  |  |  |  |  |  |  | Fluorometholone | 38 |
| 36 | N | 2 | Uveitis | Resolved | 51 | 287 | Betamethasone sodium phosphate | 35 |
|  |  |  |  |  |  |  | Fluorometholone | 56 |
| 37 | N | 2 | Iritis | Resolved | 50 | 183 | Levofloxacin | 5 |
|  |  |  |  |  |  |  | Bromfenac sodium | 8 |
|  |  |  |  |  |  |  | Betamethasone sodium phosphate, gatifloxacin | 35 |
|  |  |  |  |  |  |  | Dexamethasone sodium phosphate | 43 |
|  |  |  |  |  |  |  | Acyclovir | 51 |
| 38 | N | 1 | Iritis | Resolved | 481 | 25 | Difluprednate | 1 |
| 39 | N | 2 | Iritis | Resolved | 14 | 108 | Betnesol-N | 1 |
|  |  |  |  |  |  |  | Moxifloxacin hydrochloride | 15 |
| 40 | N | 1 | Iritis | Resolved | 145 | 369 | Levofloxacin, ofloxacin | 1 |
|  |  |  |  |  |  |  | Betamethasone sodium phosphate | 2 |
| 41 | N | 1 | Uveitis | Resolved | 12 | 214 | Betamethasone sodium phosphate, moxifloxacin hydrochloride | 15 |
| 42 | N | 1 | Iridocyclitis | Resolved | 28 | 66 | Dexamethasone, retinol | 1 |
| 43 | Y | 3 | Anterior chamber inflammation; retinal artery embolism | Not resolved | 65 | Ongoing | Neodecadron | 1 |
|  |  |  |  |  |  |  | Retinal laser coagulation | 40 |
| 44 | N | 1 | Chorioretinitis | Resolved | 56 | 57 | Neodecadron, Ster-dex | 1 |
|  |  |  |  |  |  |  | Hypromellose, dexamethasone | 23 |
|  |  |  |  |  |  |  | Cethexonium | 24 |
| 45 | N | 1 | Anterior chamber flare | Resolved | 180 | 11 | Neodecadron | 1 |
| 46 | Y | 3 | Uveitis | Resolved with sequelae | 156 | 130 | Prednisolone acetate | 1 |
| 47 | N | 1 | Eye Inflammation | Resolved | 260 | 25 | Prednisolone acetate | 1 |
| 48 | N | 1 | Iridocyclitis | Resolved | 260 | 71 | N/A | N/A |
| 49 | N | 1 | Anterior chamber cell | Resolved | 29 | 29 | N/A | N/A |
| 50 | Y | 2 | Uveitis | Resolved | 176 | 204 | Dexamethasone | 8 |
|  |  |  |  |  |  |  | Aflibercept | 29 |

^a^Severity: 1 = mild; 2 = moderate; 3 = severe. *Mild* was defined as usually transient in nature and generally not interfering with normal activities. *Moderate* was defined as sufficiently discomforting to interfere with normal activities. *Severe* was defined as preventing normal activities.

^b^*Medical Dictionary for Regulatory Activities* version 20.1 has been used for reporting.

^c^Day of the IOI-related event onset relative to the first day of the study treatment.

^d^Concomitant medication and nondrug therapies up to the adverse event end date or 56 days since the adverse event start date, whichever comes earlier. If more than 1 IOI-related adverse event was reported at the onset date of the first IOI-related adverse event, all concomitant medications and nondrug therapies on or before the latest adverse event end date are listed.

^e^Day of the concomitant medication or nondrug therapy start relative to the IOI-related adverse event onset date.

^f^Reported term.
